# Platelet-derived integrin- and tetraspanin-enriched tethers exacerbate severe inflammation

**DOI:** 10.1126/science.adu2825

**Published:** 2026-01-22

**Authors:** Charly Kusch, David Stegner, Lukas J. Weiss, Paquita Nurden, Philipp Burkard, Denise Johnson, Wolfgang Bergmeier, Ceylan Onursal, Stefano Navarro, Christian Hackenbroch, Dennis Pfeiffer, Sabrina Ivana Bonfiglio, Mara Meub, Carina Gross, Joachim Schenk, Valeria Fumagalli, Kristina Mott, Markus Bender, Matteo Iannacone, Oliver Andres, Wolfgang Kastenmüller, Katrin G. Heinze, Markus Sauer, Harald Schulze, Klaus Ley, Alan T. Nurden, Bernhard Nieswandt

**Affiliations:** 1 Institute of Experimental Biomedicine, Chair I, University Hospital Würzburg, Germany.; 2 Rudolf Virchow Center for Integrative and Translational Bioimaging, Julius-Maximilians-Universität Würzburg (JMU), Würzburg, Germany.; 3 Department of Internal Medicine I, University Hospital Würzburg, Würzburg, Germany.; 4 Institut Hospitalo-Universitaire LIRYC, Hôpital Xavier Arnozan, Pessac, France.; 5 Department of Biochemistry and Biophysics, and Blood Research Center, University of North Carolina at Chapel Hill, Chapel Hill, NC, USA.; 6 Department of Biotechnology and Biophysics, Biocenter, Julius-Maximilians-Universität Würzburg (JMU), Würzburg, Germany.; 7 Division of Immunology, Transplantation, and Infectious Diseases, IRCCS San Raffaele Scientific Institute, Milan, Italy.; 8 Vita-Salute San Raffaele University, Milan, Italy.; 9 Department of Pediatrics, University Hospital Würzburg, Würzburg, Germany.; 10 Institute of Systems Immunology, University of Würzburg, Julius-Maximilians-Universität Würzburg (JMU), Würzburg, Germany.; 11 Immunology Center of Georgia (IMMCG), Medical College of Georgia, Augusta University, Augusta, GA, USA.

## Abstract

Platelet integrin αIIbβ3 is essential for hemostasis, thrombosis, and inflammation. We found that ligation of αIIbβ3 by von Willebrand factor or fibrin under flow triggered its accumulation in plasma membrane extensions or “platelet-derived integrin- and tetraspanin-enriched tethers” (PITTs). PITTs remained anchored to leukocytes or endothelial cells, whereas the partially αIIbβ3-deficient platelet body detached. although still responsive to stimuli, αIIbβ3-deficient platelets did not support thrombus formation. PITTs promoted leukocyte activation and vascular inflammation in mouse models of infection and endotoxemia, and αIIbβ3 blockade reduced immune-mediated tissue damage. In patients with sepsis, COVID-19, or severe infections, PITT formation and platelet αIIbβ3 loss correlated with disease severity and adverse outcomes. We propose that PITTs are proinflammatory structures that amplify immune responses while contributing to platelet dysfunction in thrombo-inflammatory disease.

## Abstract (summary)

**INTRODUCTION:**

Integrins are a family of heterodimeric transmembrane receptors that link the extracellular matrix to the cytoskeleton and regulate adhesion, migration, and signaling in many cell types. The platelet integrin αIIbβ3 (also designated GPIIb/IIIa) is among the best-studied integrins and is indispensable for hemostasis and thrombosis. Beyond their established role in clotting, platelets contribute to immune-mediated tissue damage in inflammatory and infectious disease.

**RATIONALE:**

Because the platelet effector mechanisms that mediate thrombo-inflammation were unclear, we sought to understand the role of αIIbβ3 and how its engagement contributes to altered platelet function.

**RESULTS:**

In blood smears and tissue samples from patients with severe COVID-19, sepsis, or bacterial infection, we observed alterations in platelet morphology and integrin expression. Specifically, we noted the presence of elongated platelet-derived membrane tethers that stained strongly for αIIbβ3 and its associated tetraspanin CD9. We called these structures platelet-derived integrin- and tetraspanin-enriched tethers (PITTs). Indicators of an increased abundance of PITT formation correlated with reduced αIIbβ3/CD9 surface levels in circulating platelets. Flow cytometry confirmed selective loss of these receptors, whereas other major glycoproteins were preserved. Using human and murine platelets, we demonstrated that ligation of αIIbβ3 by von Willebrand factor or fibrin under flow was sufficient to trigger rapid integrin clustering and tether formation, independent of classical platelet activation. PITTs remained attached to leukocytes or endothelium, whereas the platelet body detached, partially depleted of αIIbβ3/CD9. We generated *Itga2b-GFP* (green fluorescent protein) knock-in mice and performed live imaging of PITT formation in vitro and confirmed αIIbβ3/CD9 enrichment in PITTs and their detachment from platelet bodies and deposition in inflamed lungs during endotoxemia, bacterial pneumonia, or SARS-CoV-2 infection using in vivo mouse studies.

PITTs formed without detectable platelet degranulation, phosphatidylserine exposure, or integrin activation, distinguishing them from classical extracellular vesicles or activation-induced protrusions. In vivo, PITTs decorated neutrophils, inducing calcium fluxes and up-regulation of activation markers, suggesting that PITTs could promote inflammatory signaling. In models of acute lung injury, genetic deletion of von Willebrand factor or pharmacologic blockade of αIIbβ3 decreased PITT formation, neutrophil infiltration, and tissue damage. In patients with sepsis, reduced platelet CD41a expression—reflecting PITT release—correlated with higher sequential organ failure assessment (SOFA) scores, acute respiratory distress syndrome, and mortality. Stratification by CD41a levels revealed that patients in the lowest quartile had substantially increased odds for adverse outcomes.

**CONCLUSION:**

We propose that PITTs act as platelet-derived structures that redistribute the αIIbβ3/CD9 pool during severe infection and inflammation. By anchoring to leukocytes and endothelial cells, PITTs may amplify vascular inflammation and immune activation while leaving behind platelets with impaired adhesive function. This mechanism may explain the paradoxical coexistence of thrombo-inflammation and bleeding in critical illness. Beyond hemostasis, αIIbβ3 may serve as a proinflammatory effector that shapes immune responses and clinical outcome in inflammatory and infectious disease. Targeting PITT formation may offer new opportunities to modulate thrombo-inflammation without impairing platelet viability.

## Editor’s summary

Altered platelet function has been proposed to contribute to the dysregulated blood clotting and inflammation that occur during extreme immune responses. Kusch *et al*. observed that platelets from patients with severe COVID infection or sepsis had a distinctive morphology, with long membrane extensions that were enriched in an adhesion molecule, the integrin #IIb#3 (see the Perspective by Perrella and Rayes). In vitro, murine platelets formed these structures, described as platelet-derived integrin and tetraspanin-enriched tethers (PITTs), under flow when #IIb#3 was triggered. In mouse models of lung inflammation, PITTs separated from the platelet body and remained attached to endothelial cells and neutrophils, stimulating inflammation. Detachment of PITTs left platelets depleted of #IIb#3 and impaired in their ability to promote blood clots. In humans, platelets with indicators of PITT formation were correlated with worse disease severity. —Sarah H. Ross

## Graphical Abstract

**Nonactivated platelets release integrin- and tetraspanin-enriched tethers to promote thrombo-inflammation.** Circulating platelets can use their principal adhesion receptor, integrin αIIbβ3, to switch between hemostatic and thrombo-inflammatory programs. At sites of vascular injury, classical activation triggers inside-out signaling of αIIbβ3, promoting fibrinogen binding, platelet adhesion, and thrombus formation. Under thrombo-inflammatory conditions, nonactivated platelets generate PITTs that can attach to leukocytes and endothelial cells, exacerbating inflammation, whereas the platelet body detaches in a state partially depleted of αIIbβ3. Rest., resting; act., activated. [Figure created with BioRender.com]

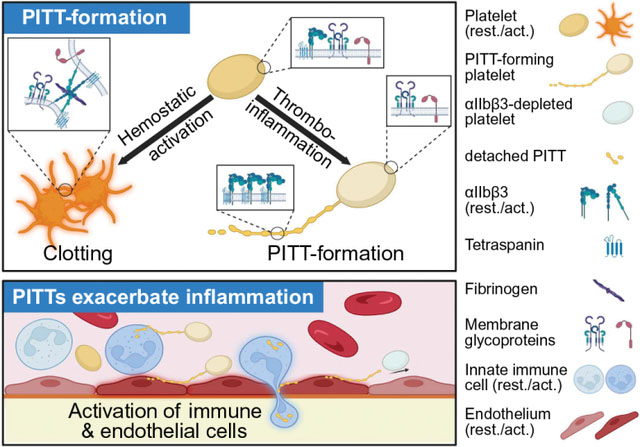

Integrins are a ubiquitously expressed family of heterodimeric transmembrane receptors that connect the extracellular matrix (ECM) to the cytoskeleton and serve as bidirectional signaling molecules that regulate a wide range of cellular functions, including cell adhesion, migration, spreading, differentiation, proliferation, and apoptosis (1). Among the 24 known integrins in mammals, αIIbβ3 (also known as GPIIb/IIIa and CD41/CD61), the major integrin of blood platelets (~50,000 to 100,000 copies per platelet) (2), is paradigmatic, with studies of its structure and function having provided seminal insights into the biology of the entire integrin family (3).

Platelets are small, anucleate blood cells essential for vascular surveillance, hemostasis, and clot formation. Upon vascular injury, they bind to exposed ECM via glycoproteins Ib-IX (GPIb-IX) and GPVI, followed by activation and degranulation (4, 5). Soluble agonists, such as adenosine 5′-diphosphate (ADP), thromboxane A_2_ (TxA_2_), and thrombin, amplify activation and the shift of αIIbβ3 from a low- to a high-affinity state by “inside-out signaling” to mediate firm adhesion and aggregation (6–8). Ligand binding also induces αIIbβ3 clustering and “outside-in signaling,” promoting actin reorganization and force generation. The latter are critical for stable platelet adhesion, thrombus consolidation, and clot retraction (3, 9, 10). The critical role of αIIbβ3 in hemostasis is demonstrated by Glanzmann thrombasthenia (GT), a bleeding disorder caused by absent or dysfunctional αIIbβ3 (3, 11, 12).

Emerging evidence further supports a critical role for αIIbβ3 and platelets in thrombo-inflammatory states, but the underlying mechanisms remain poorly understood (13–16). In particular, there is evidence for altered platelet function in pulmonary inflammation and acute respiratory distress syndrome (ARDS), whose symptoms are hallmarks of severe COVID-19 caused by severe acute respiratory syndrome coronavirus 2 (SARS-CoV-2) infection. Indeed, experimental and clinical data point to a role of platelets in fueling pulmonary inflammation in this setting (17–20). In this work, we aimed to delineate the platelet effector mechanisms driving this inflammatory response and to understand how their activation is linked to altered platelet function.

## Results

### Loss of αIIbβ3/CD9 in circulating platelets of patients with critical infection

**Fig. 1.**
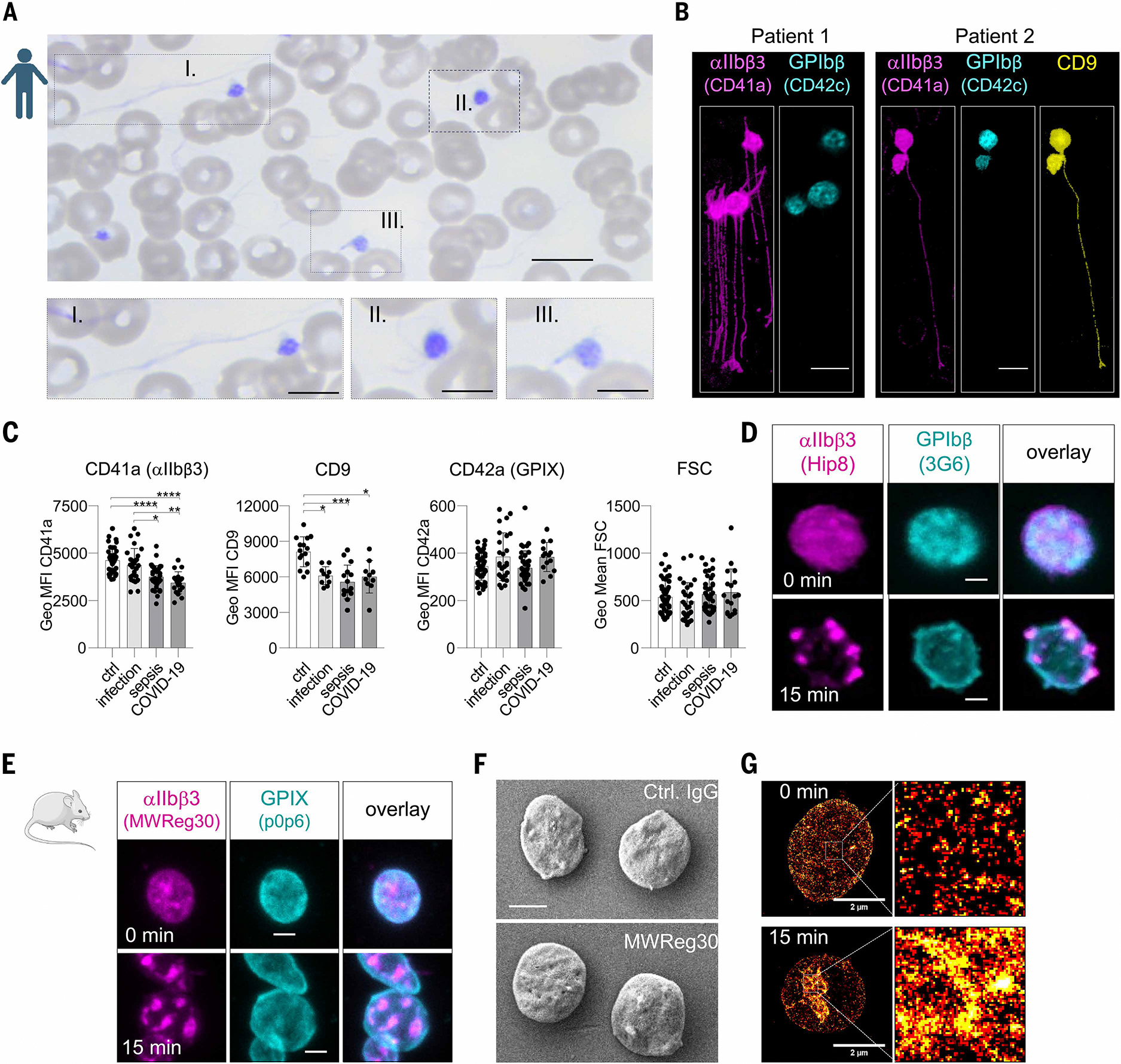
Loss of αIIbβ3/CD9 in circulating platelets of patients with critical infection. (**A**) May-Grünwald-Giemsa–stained blood smear from a critically ill COVID-19 patient showing pronounced tether formation by apparently nonactivated platelets. Such tethers were absent in smears from healthy controls. Scale bar: 10 μm. (**B**) Detection of long αIIbβ3^+^/GPIbβ^−^ tethers in blood smears from two critically ill COVID-19 patients using confocal fluorescence microscopy. In patient 2, tethers also stained positive for CD9. Scale bar: 10 μm. (**C**) Selective loss of αIIbβ3 and CD9 in circulating platelets from patients with critical inflammatory conditions. Surface abundance of CD41a (αIIbβ3), CD9, and CD42a (GPIX), as well as forward scatter (FSC), was analyzed by flow cytometry in platelets from patients with infection, sepsis, or COVID-19, and compared to healthy controls (ctrl). Gating strategy is shown in fig. S1B. Statistical analysis: Kruskal-Wallis test. Data are shown as mean ± SD. Each datapoint represents one individual (*n* = 10 to 39). *P < 0.05, **P < 0.01, ***P < 0.001, ****P < 0.0001. (**D**) Maximum intensity projections of confocal images showing αIIbβ3 and GPIbβ (p0p1^AF488^) distribution in human platelets fixed either before (0 min) or after 15 min of incubation with anti-αIIbβ3 mAb Hip8^AF647^. Pronounced αIIbβ3 clustering induced by Hip8 is apparent. Scale bar: 1 μm. (**E**) Maximum projections of confocal images comparing αIIbβ3 and GPIX (p0p6) distribution in mouse platelets fixed before (0 min) or after 15 min of incubation with MWReg30^AF647^. Again, prominent αIIbβ3 clustering can be seen. Scale bar: 1 μm. (**F**) Scanning electron microscopy images of mouse platelets treated with control IgG or MWReg30 (10 μg/ml). Platelets showed no signs of activation under either condition. Scale bar: 2 μm. (**G**) *d*STORM images of mouse platelets treated with MWReg30^AF647^as in (E), confirming αIIbβ3 integrin clustering at the molecular level, compared to the homogeneous distribution in untreated platelets. Scale bar: 2 μm.

When analyzing platelet morphology on blood smears from critically ill patients with COVID-19 (table S1) by light microscopy, we observed abundant membrane tethers of varying lengths (up to 30 μm) in ~40% of samples. In several cases, these tethers were detached from the platelet body (Fig. 1A). Immunofluorescence staining revealed that the tethers were strongly positive for αIIb (CD41a), whereas GPIbβ (CD42c) was absent (Fig. 1B). In the membrane, αIIbβ3 associates with the tetraspanin CD9, which regulates its localization and supports activation (21, 22). Costaining confirmed the presence of CD9 in the tethers (Fig. 1B and fig. S1). These structures were essentially absent in healthy controls (fig. S1A), suggesting that their formation was a specific response to severe illness in COVID-19. Given that platelet adhesion to glass is primarily mediated by αIIbβ3 (23), we hypothesize that tether formation was mechanically driven via anchored αIIbβ3, resulting in the segregation of αIIbβ3/CD9-rich tethers from the platelet. We called these elongated membrane structures platelet-derived integrin- and tetraspanin-enriched tethers (PITTs).

We hypothesized that if PITTs formed in sufficient numbers and detached, platelets would be depleted of αIIbβ3 and CD9 in the patients. We analyzed the levels of receptors on the surface of platelets isolated from critically ill COVID-19 patients by flow cytometry and found that αIIbβ3 (CD41a) and CD9 were reduced in number, whereas CD42a (GPIX) levels remained unchanged compared with 47 healthy controls (Fig. 1C). Despite the partial loss of αIIbβ3 and CD9, these platelets were in an apparently resting state, because binding of the αIIbβ3 activation reporter PAC-1 and exposure of the CD62P surface were nearly absent (fig. S1C) (24). This finding prompted us to extend our studies to patients with infection or sepsis (table S1). In both cohorts, circulating platelets had lower αIIbβ3 and CD9 expression, whereas CD42a levels were comparable to those of healthy controls (Fig. 1C). To illustrate the concurrent loss of CD41a and CD9, we performed flow cytometric dot plot analyses, confirming parallel reduction of both markers across all patient groups (fig. S1D). Moreover, the forward scatter signal, an indicator of cell size, was not different, suggesting that platelet morphology was preserved (Fig. 1C). We also observed numerous PITTs in blood smears of sepsis patients (fig. S1E). These data are therefore consistent with a partial loss of αIIbβ3/CD9 from platelets during severe infection and inflammation, conditions that have been associated with aberrant platelet function (25–27).

### High lateral mobility of αIIbβ3 in the platelet membrane

The selective enrichment of αIIbβ3 in PITTs suggested a high lateral mobility of the integrin within the platelet membrane. To investigate this, we incubated human platelets with Hip8^AF647^, a fluorophore-coupled monoclonal antibody (mAb) to αIIbβ3, and assessed clustering of the integrin as a readout of its mobility in the membrane. Although Hip8 did not activate platelets, it induced αIIbβ3 clustering and accumulation in distinct membrane areas on the cell surface (capping) (Fig. 1D), confirming previous observations (28, 29). Similar effects were seen with another anti-αIIbβ3 mAb (fig. S1E), whereas mAbs against GPIbβ (Fig. 1D), GPV, or GPIbα did not induce receptor clustering (fig. S1F).

To enable mechanistic studies, we extended these findings to murine platelets. Incubation with anti-αIIbβ3 mAb MWReg30^AF488^ (30) led to prominent αIIbβ3 clustering (Fig. 1E) without inducing cellular activation (Fig. 1F and fig. S2, A and B). Super-resolution *d*STORM imaging (31) showed αIIbβ3 localized in discrete membrane patches, with large areas devoid of signal (Fig. 1G). We also did not detect major alterations in actin and tubulin cytoskeletal architecture (fig. S2C). Similar clustering occurred with other anti-αIIbβ3 mAbs (32) (fig. S2D), whereas mAbs against GPIX (Fig. 1E), GPIbα, GPIbβ, GPV, or GPVI did not induce clustering (fig. S2D). Consistent with this, other abundantly expressed platelet receptors, including GPIb-IX-V subunits, GPVI, and CD62P, were absent from PITTs despite robust staining on the platelet body (fig. S2E).

We found that αIIbβ3 clustering occurred within seconds of MWReg30 binding (movie S1). The small GTP-binding protein Arf6 has been implicated in αIIbβ3 trafficking and endocytosis (33). However, MWReg30-induced αIIbβ3 clustering was indistinguishable between Arf6-deficient (*Arf6*^*fl/fl-Pf4 cre*^) and control platelets, even in the presence of the clathrin inhibitor Pitstop 1 (fig. S2F and movie S2). MWReg30 remained detectable on the surface of nonfixed wild-type (WT) as well as Arf6-deficient platelets throughout the observation (60 min; fig. S2G).

### Rapid and highly specific recruitment of αIIbβ3 into extending tethers

**Fig. 2.**
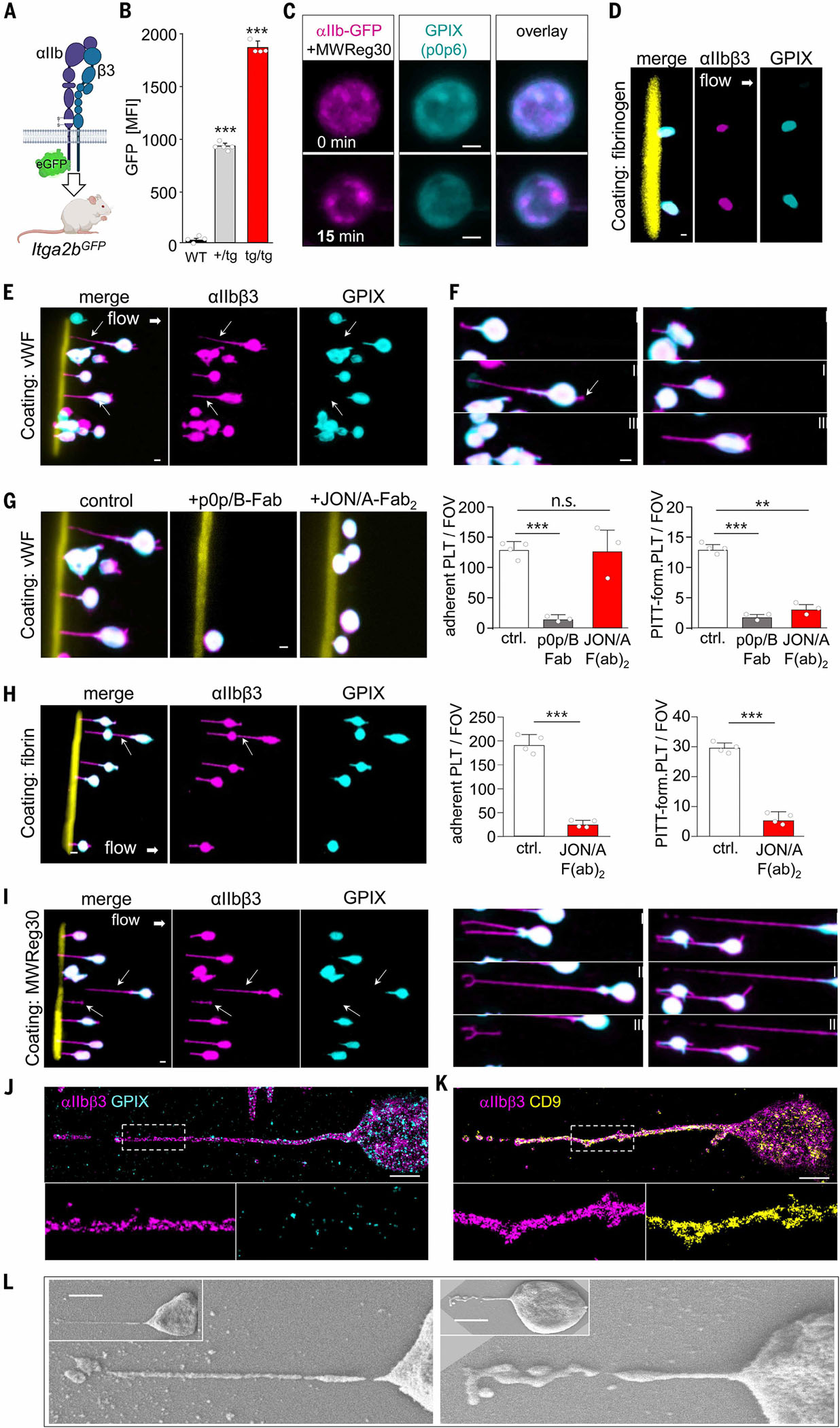
Formation of platelet-derived integrin- and tetraspanin-enriched tethers (PITTs). (**A** to **C**) Generation of a mouse line expressing GFP-tagged integrin αIIb (CD41). (A) Schematic representation of the GFP-tagged integrin. (B), Flow cytometric detection of αIIb^GFP^ in WT, heterozygous (tg/+), and homozygous (tg/tg) *Itga2b*^*GFP*^ mice (for gating strategy, see fig. S3E). Each data point represents one mouse (*n* = 4 to 6; Welch’s *t* test with Bonferroni correction). Data are shown as mean ±SD; ***P < 0.001. (C), Maximum intensity projections of confocal images showing the distribution and clustering of αIIb^GFP^β3 and GPIX (p0p6^AF647^) in platelets fixed before (0 min) or after 15 min of incubation with MWReg30 (10 μg/ml). (**D**) Representative image of *Itga2b*^*GFP/GFP*^ platelets counterstained with anti-GPIX^AF546^ during a flow chamber run over fibrinogen-coated (yellow) PRIMO patterns at a shear rate of 1000 s^−1^. Arrow indicates direction of flow (left to right). (**E**) Representative images of *Itga2b*^*GFP/GFP*^ platelets counterstained with anti-GPIX^AF647^ during a flow chamber run over vWF-coated PRIMO patterns at 1000 s^−1^. Selective accumulation of αIIbβ3 (magenta), but not GPIX (cyan), can be seen in the tethers. (**F**) Insets of flow chamber runs depicted as time series highlighting PITT formation. (**G**) Representative images of *Itga2b*^*GFP/GFP*^ platelets (anti-GPIX^AF647^) during flow chamber runs over vWF-coated PRIMO patterns at 1000 s^−1^, treated with control Fab (left; see also movie S3), function-blocking anti-GPIbα (p0p/B-Fab, middle, movie S4), or anti-αIIbβ3 (JON/A-F(ab)_2_ (right, movie S5) (10 μg/ml each). Right panels: Quantitative analysis of platelet adhesion (left) and PITT formation (right) of *Itga2b*^*GFP/GFP*^ platelets on vWF in the presence of vehicle (cntrl), anti-GPIbα (p0p/B-Fab), or anti-αIIbβ3 (JON/A-F(ab)_2_. Data are shown as mean ± SD, *n* = 3 to 4 mice [representative of three independent experiments; one-way analysis of variance (ANOVA), Bonferroni corrected]. n.s., not significant, *p<0.05, **p<0.01, ***p<0.001. (**H**) Representative images of *Itga2b*^*GFP/GFP*^ platelets counterstained with anti-GPIX^AF546^ during a flow chamber run over fibrin-coated PRIMO patterns at a shear rate of 1000 s^−1^. Right: Quantitative analysis of platelet adhesion (left) and PITT formation (right) on fibrin in the presence of vehicle or anti-αIIbβ3 (JON/A-F(ab)_2_. Data shown as mean ±SD, n=4 mice (representative of 3 independent experiments; Welch’s *t* test). (**I**), Representative images of *Itga2b*^*GFP/GFP*^ platelets counterstained with anti-GPIX^AF647^ during a flow chamber run over MWReg30-coated PRIMO patterns at 1000 s^−1^. Right: Time series highlighting different PITT-forming and -releasing platelets (movies S6 and S7), showing PITT/*d*PITT formation. White arrows indicate PITT formation in the direction of flow. (**J** and **K**) *d*STORM images of fixed platelets and PITTs formed on MWReg30-coated PRIMO patterns stained for αIIbβ3 (JON6^AF647^, magenta) and GPIX (p0p6^CF®568^, cyan) (J) or αIIbβ3 (JON6^CF®568^, magenta) and CD9 (ULF1^AF647^, yellow) (K). (**L**) Scanning electron microscopy showing a long protrusion extending from the parent platelet (left) and a second example in which the tether has fractured (right). Insets show discoid parent platelets at lower magnification. Images acquired on MWReg30-coated PRIMO patterns. (C to L) Scale bar: 1 μm.

To study αIIbβ3 mobility and segregation into PITTs without antibody-induced clustering, we generated a mouse line expressing a green fluorescent protein (GFP)–tagged αIIb (*Itga2b-GFP*). The tagged integrin was readily detectable in circulating platelets by flow cytometry, fluorescence microscopy, and Western blot analysis (Fig. 2, A to C, and fig. S3, A and B). *Itga2b*^*GFP/GFP*^ platelets responded normally to agonists and fully spread on fibrinogen (fig. S3, C and D), suggesting that integrin signaling was not disrupted by the GFP tag (29). Incubation with MWReg30 (5 μg/ml) induced clustering and capping of the integrin (Fig. 2C), confirming high lateral mobility of αIIb^GFP^β3.

Using these mice, we sought to test whether resting platelets formed PITTs on surface-bound ligands in the absence of agonist stimulation. For this task, we used the PRIMO maskless ultraviolet (UV)–patterning system (Alvéole, Paris, France) to generate 2 × 40-μm adhesion fields. We coated the micropatterns with von Willebrand factor (vWF) or fibrinogen and perfused them with blood from *Itga2b*^*GFP/GFP*^ mice at a rate of 1000 s^−1^. Only a few platelets adhered to fibrinogen, and tether formation was nearly undetectable (Fig. 2D). By contrast, vWF-coated patterns supported robust platelet adhesion and frequent formation of shear-resistant αIIb^GFP+^/GPIX^−^ tethers. PITTs formed at the rear edge of the platelets, but αIIb^GFP+^/GPIX^−^ tethers also transiently extended to the front in the direction of flow (Fig. 2, E and F, and movie S3).

Under high shear, platelet translocation on vWF is mediated by GPIbα (34), an interaction previously reported to induce the formation of thin-membrane tethers in a process that is shear dependent (35). In our assay, blocking the GPIbα-vWF interaction with an anti-GPIX antibody, p0p/B^Fab^ (15), abolished platelet adhesion and PITT formation (Fig. 2G and movie S4). By contrast, the anti-αIIbβ3 JON/A-F(ab)_2_ (36), which blocks the αIIbβ3-vWF interaction, reduced PITT formation, without affecting platelet tethering to vWF (Fig. 2G and movie S5).

At sites of injury or inflammation, thrombin converts fibrinogen to fibrin, which strongly promotes thrombo-inflammation, a process recently shown to be important for the pathophysiology of severe COVID-1 9 infection (37). Fibrin potently induced the formation of PITTs that were overall longer (up to 20 μm) than those observed on vWF and frequently also appeared to connect two platelets (Fig. 2H). Blockade of αIIbβ3 by JON/A-Fab_2_ strongly inhibited platelet attachment and PITT formation on fibrin (Fig. 2H). These results suggested that engagement and/or anchoring of αIIbβ3, but not GPIbα, triggered PITT formation.

To test whether αIIbβ3 engagement alone was sufficient to induce PITT formation, we coated micropatterns with MWReg30 and perfused blood from *Itga2b*^*GFP/GFP*^ mice at a flow rate of 1000 s^−1^. Platelets were rapidly captured and extended long PITTs at the rear, sometimes detaching and leaving behind detached PITTs (*d*PITT) (Fig. 2I and movies S6 and S7). As we observed for platelets attaching to vWF, transient front-facing αIIb^GFP+^/GPIX^−^ tethers also formed but retracted quickly—likely owing to lack of anchorage. αIIbβ3 and CD9 were enriched in (*d*)PITTs, whereas GPIb-IX and other prominent surface receptors were minimally present (Fig. 2, J and K). Scanning electron microscopy confirmed the presence of *d*PITTs and that PITT-forming platelets continued to be discoid, indicating that they were in a resting state (Fig. 2L).

We inhibited “classical” platelet activation pathways by combinations of either EGTA (0.5 mM)–BAPTA-AM (20 μM) or ASA (300 μM)–apyrase (0.02 U/ml)–prostaglandin I_2_ (PGI_2_, 0.1 μg/ml) and found that it did not impair PITT formation on fibrin or MWReg30 (fig. S4). PITTs did not stain for the high-affinity conformation of αIIbβ3 [JON/A^PE^ (36)], CD62P, or phosphatidylserine, which distinguished them from classical extracellular vesicles, such as microparticles and exosomes (38–40). Likewise, they were distinct from shear-induced platelet tethers (35, 41) and flow-induced protrusions (42), which express GPIb-IX and activation markers. We did not observe PITT formation in flow adhesion experiments on thrombogenic surfaces (collagen)—i.e., conditions that favor platelet activation and thrombus formation (fig. S4C).

### Circulating platelets can lose their entire integrin αIIbβ3 pool

**Fig. 3.**
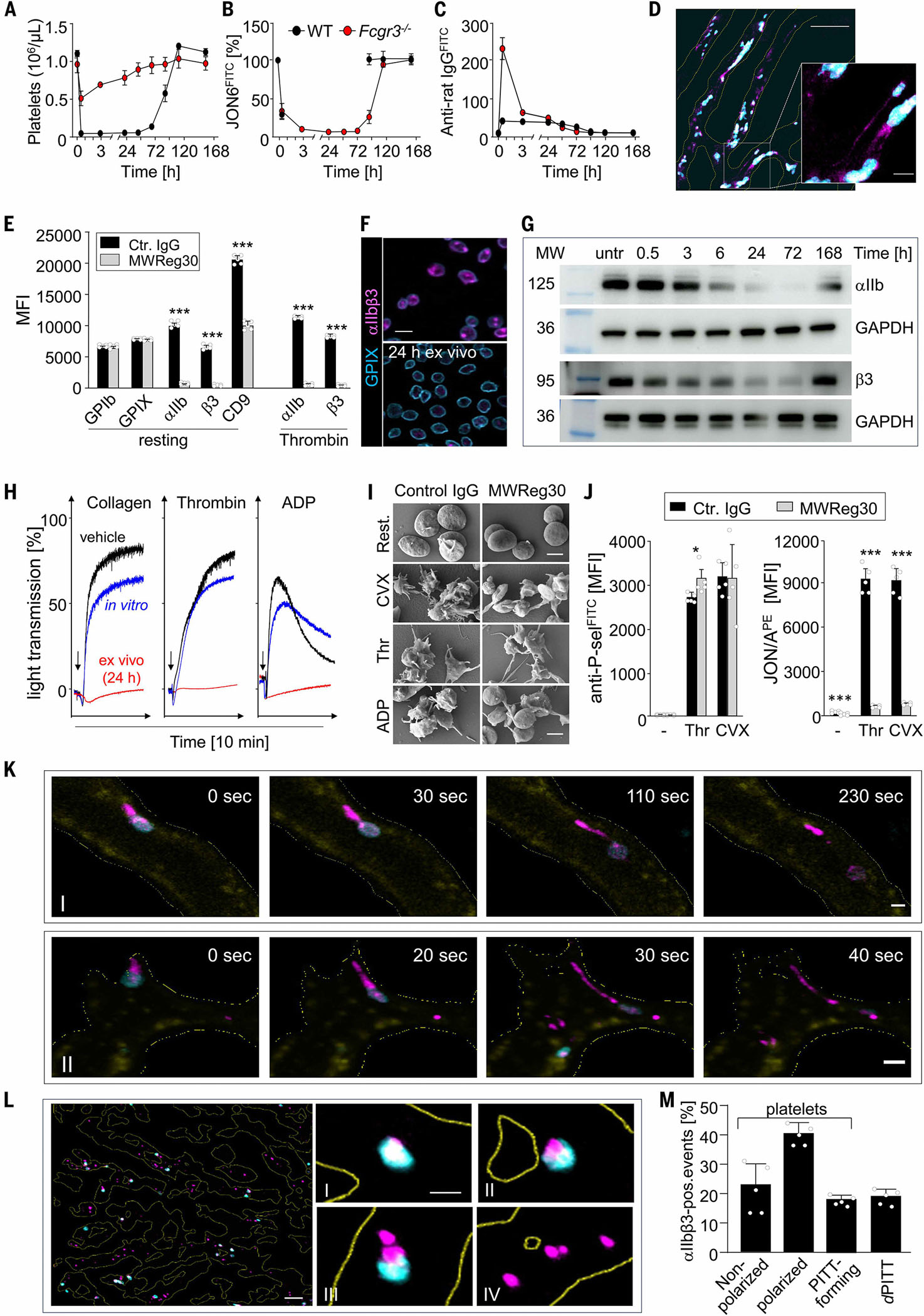
Anchoring of ligated integrin αIIbβ3 in mouse models results in its disengagement from the platelet body and deposition in long tethers. (**A**) Platelet counts in WT (black) and *Fcgr3*^*−/−*^ mice (red) were monitored by flow cytometry after intravenous injection of MWReg30 (3 μg/g BW). (**B** and **C**) Surface expression of αIIbβ3 (B) and bound MWReg30 (C) was assessed by flow cytometry using JON6^FITC^ and anti-rat IgG^FITC^ antibodies, respectively. (A to C) Gating strategy and representative plots are shown in fig. S3E. Data are presented as mean ± SD (*n* = 6 mice; representative of two independent experiments). (**D**) WT mice were treated with MWReg30^AF488^ (0.2 μg/g i.v.) and liver sinusoids were imaged immediately by CLSM. Platelets were counterstained with anti-GPIX^AF546^ (0.2 μg/g), and the vessel bed (stained with anti-CD105^AF647^) is highlighted by yellow lines. Snapshot from a representative video (movie S8) showing platelet accumulation and attachment to LSECs 4 min after MWReg30^AF488^ injection. Widespread formation of αIIbβ3^+^/GPIX^−^ tethers on the LSEC surface is visible. Scale bar: 2 μm. (**E**) Flow cytometric detection of the indicated surface glycoproteins in *Fcgr3*^*−/−*^ mice 24 hours after injection of control IgG or MWReg30 (3 μg/g BW each). Diluted whole blood was incubated with FITC-labeled mAbs at saturating concentrations, and mean fluorescence intensity (MFI) was measured. Where indicated, platelets were activated with thrombin (0.01 units/ml). *n* = 5 mice; representative of three independent experiments. ***P < 0.001. (**F**), Maximum intensity projections of confocal images of platelets from *Fcgr3*^*−/−*^ mice after injection of MWReg30^AF488^ (3 μg/g BW i.v.; magenta). Platelets were fixed and costained with anti-GPIX^AF546^ (10 μg/ml for 15 min; cyan). Scale bar: 5 μm. (**G**) Western blot analysis of αIIb (CD41) and β3 (CD61) expression in platelet lysates from *Fcgr3*^*−/−*^ mice at the indicated time points after MWReg30 injection (3 μg/g BW). GAPDH served as loading control. Untr, untreated (for quantification and uncropped blots, see fig. S6, B and C). (**H**) Platelets were incubated with MWReg30 (10 μg/ml) for 10 min in vitro or isolated 24 hours after MWReg30 (3 μg/g BW) injection in *Fcgr3*^*−/−*^ mice, then stimulated with the indicated agonists (black arrow) in an aggregometer. Light transmission was recorded for 10 min. (**I**) Scanning electron microscopy images of platelets from *Fcgr3*^*−/−*^ mice 24 hours after injection of 3 μg/g BW control IgG or MWReg30. Platelets were left untreated (rest) or stimulated with convulxin (CVX, 0.5 μg/ml), thrombin (Thr, 0.01 U/ml), or 10 μM ADP for 15 min. Scale bar: 1 μm. (**J**) Washed platelets from *Fcgr3*^*−/−*^ mice 24 hours after injection of control IgG or MWReg30 (3 μg/g BW) were left untreated or stimulated with thrombin (0.1 U/ml) or convulxin (CVX, 0.5 μg/ml). Platelet activation was assessed by flow cytometric detection of P-selectin exposure (anti-P-selectin^FITC^) and αIIbβ3 activation (JON/A^PE^). *n* = 5 mice, representative of three independent experiments. *P < 0.05; ***P < 0.001. (**K**) WT platelets were double labeled in vitro with MWReg30^AF488^ and anti-GPIX^AF546^ (5 μg/ml for 10 min) and transfused into *Fcgr3*^*−/−*^ mice, and liver sinusoids were visualized immediately by CLSM. The vessel bed (stained with anti-CD105^AF647^) is highlighted by dashed yellow lines. Snapshots from two representative videos (movies S9 to S11) show platelets forming αIIbβ3-enriched tethers on LSECs, flowed by detachment of αIIbβ3-depleted platelet into circulation. Scale bar: 2 μm. (**L**) Fluorescence micrographs of liver cryosections from *Fcgr3*^*−/−*^ mice 30 min after infusion of WT platelets prestained with MWReg30^AF488^ (magenta) and anti-GPIX^AF546^ (cyan) (5 μg/mL each for 5 min). Dashed yellow lines indicate the endothelial border. Scale bar: 5 μm. The different phases of PITT formation are illustrated: (I) nonpolarized, (II) polarized, (III) PITT-extending platelet, and (IV) *d*PITTs. Scale bar: 5 μm and 1 μm (insets). (**M**) Quantification of nonpolarized, polarized, PITT-forming platelets, and *d*PITTs in liver cryosections from *Fcgr3*^*−/−*^ mice 30 min after platelet transfusion. In (E), (J), and (M), individual data points show data from one mouse, bars are mean ± SD; *n* = 5 mice.

To investigate PITT formation in vivo, we treated mice with MWReg30 and monitored platelet sequestration. This treatment induces rapid platelet clearance and an Fc-dependent anaphylactic reaction, circulatory collapse, and lung injury (30). A hallmark of this pathology is the accumulation of platelets and αIIbβ3-containing immune complex–like structures in organs such as liver and spleen (43). Injection of MWReg30 [3 μg per gram of body weight (BW)] in WT mice caused rapid platelet accumulation in the liver (fig. S5A) and severe thrombocytopenia, with platelet counts dropping below 3% within 30 min and remaining low for at least 48 hours (Fig. 3A).

To visualize the antibody-opsonized platelets in situ, we performed intravital confocal laser scanning microscopy (IV-CLSM). Mice were injected with anti-GPIX^AF546^ (0.22 μg/g) and anti-CD105^AF647^ (0.4 μg/g) to label platelets and liver sinusoidal endothelial cells (LSECs) (44), followed by MWReg30^AF488^ (0.2 μg/g BW). Almost immediately, MWReg30-opsonized platelets accumulated at LSECs and exhibited rapid polarization, with αIIbβ3 clustering at the “rear edge” of the cell body (relative to blood flow direction; see movie S8). These platelets extended long GPIX-negative tethers enriched in αIIbβ3 (i.e., PITTs) at the LSEC contact site (Fig. 3B; fig. S5, B and C; and movie S8).

Fcγ receptor (FcγR) III has a critical role in the clearing of immunoglobulin G (IgG)–opsonized platelets in mice (30). To study the phenotype of platelets after PITT release, we therefore used *Fcgr3*^*−/−*^ mice that are deficient in FcγRIII. In these animals, MWReg30-opsonized platelets were initially recruited to the liver (fig. S5), but platelet counts dropped only transiently and recovered to >80% within 6 hours (Fig. 3A) (30). Yet, despite the partial recovery of platelet numbers, recirculating platelets exhibited a rapid and progressive loss of surface αIIbβ3 with >70% and >95% of the receptor being lost at 3 hours and 24 hours, respectively (Fig. 3B), whereas MWReg30 binding declined to <2% of control (Fig. 3C). CD9 levels decreased by ~50% at 24 hours (Fig. 3E and table S2), with both αIIbβ3 and CD9 returning to baseline levels after ~7 days, likely via de novo platelet production. By contrast, the levels of other surface receptors (GPIb-IX-V, GPVI, CLEC-2, CD84, β1 integrins) remained largely unaltered at 24 hours (table S2). Transmission electron microscopy further confirmed that platelets recirculating after αIIbβ3 loss remained in a resting state, displaying normal granule distribution and no pseudopod formation (fig. S6A).

αIIbβ3 was nearly absent from plasma membrane and internal stores at day 1 (Fig. 3, E and F). The expression of αIIb and β3 was reduced for up to 5 days, with normalization by day 7 (Fig. 3G and fig. S6, B and C), suggesting irreversible integrin loss via PITT release. Consistently, these platelets failed to aggregate in response to collagen, thrombin, or ADP (Fig. 3H). The αIIbβ3-deficient platelets still underwent shape change (Fig. 3I) and degranulation (Fig. 3J), whereas activated αIIbβ3 (JON/A^PE^ binding) was absent (Fig. 3J). Functionally, this led to severely prolonged tail bleeding and abolished arterial thrombus formation in an FeCl_3_ injury model (fig. S6, D to F); nonetheless the platelet life span remained largely unaffected (fig. S6G), similar to the phenotype observed in *Itgb3*^*−/−*^ mice, a murine model of GT (45).

Kupffer cells in liver sinusoids contribute to the clearing of IgG-opsonized platelets in a FcγRIII-dependent manner (30). Because this mechanism overlays and possibly interferes with MWReg30-induced PITT formation, we performed liver IV-CLSM in *Fcgr3*^*−/−*^ mice to visualize the process in the absence of clearing mechanisms. WT platelets were labeled with anti-GPIX^AF546^ and MWReg30^AF488^ in vitro (5 μg/ml, 10 min), washed, and injected into *Fcgr3*^*−/−*^ recipients, previously given anti-CD105^AF647^ mAb (0.4 μg/g). Opsonized platelets rapidly adhered to LSECs, polarized with dense αIIbβ3 clusters at the contact site, and formed large PITTs (αIIbβ3^+^ GPIX^−^, up to 30 μm). The bulk of the platelet, which was almost devoid of αIIbβ3 but homogeneously stained for GPIX, then detached and returned to the circulation, typically within ~5 min (movies S9 to S11 and Fig. 3K). Of note, αIIbβ3/MWReg30 clusters were also seen in free-flowing platelets, but PITTs formed only upon attachment. Separated PITTs contracted and/or moved along the vessel wall, frequently opposite to the direction of blood flow (movie S9). Whether this was due to active migration, or transport by LSEC, is unclear. Quantitative analysis of liver cryosections confirmed that most platelets attached to LSECs were polarized and in different stages of PITT formation and that *d*PITTs were abundant (Fig. 3, L and M). In all platelets, GPIX staining remained homogeneous along the plasma membrane.

We hypothesized that anti-αIIbβ3–opsonized platelets anchor to LSECs through FcγRIIB, a scavenger receptor abundantly expressed in LSECs (46, 47). Indeed, *Fcgr3:Fcgr2b*–double knockout (DKO) mice failed to recruit platelets to the liver (fig. S7A) and did not down-regulate αIIbβ3 after MWReg30 treatment, in contrast to *Fcgr3*^*−/−*^ mice (fig. S7, B to E).

### Platelets locally deposit PITTs at sites of inflammation

**Fig. 4.**
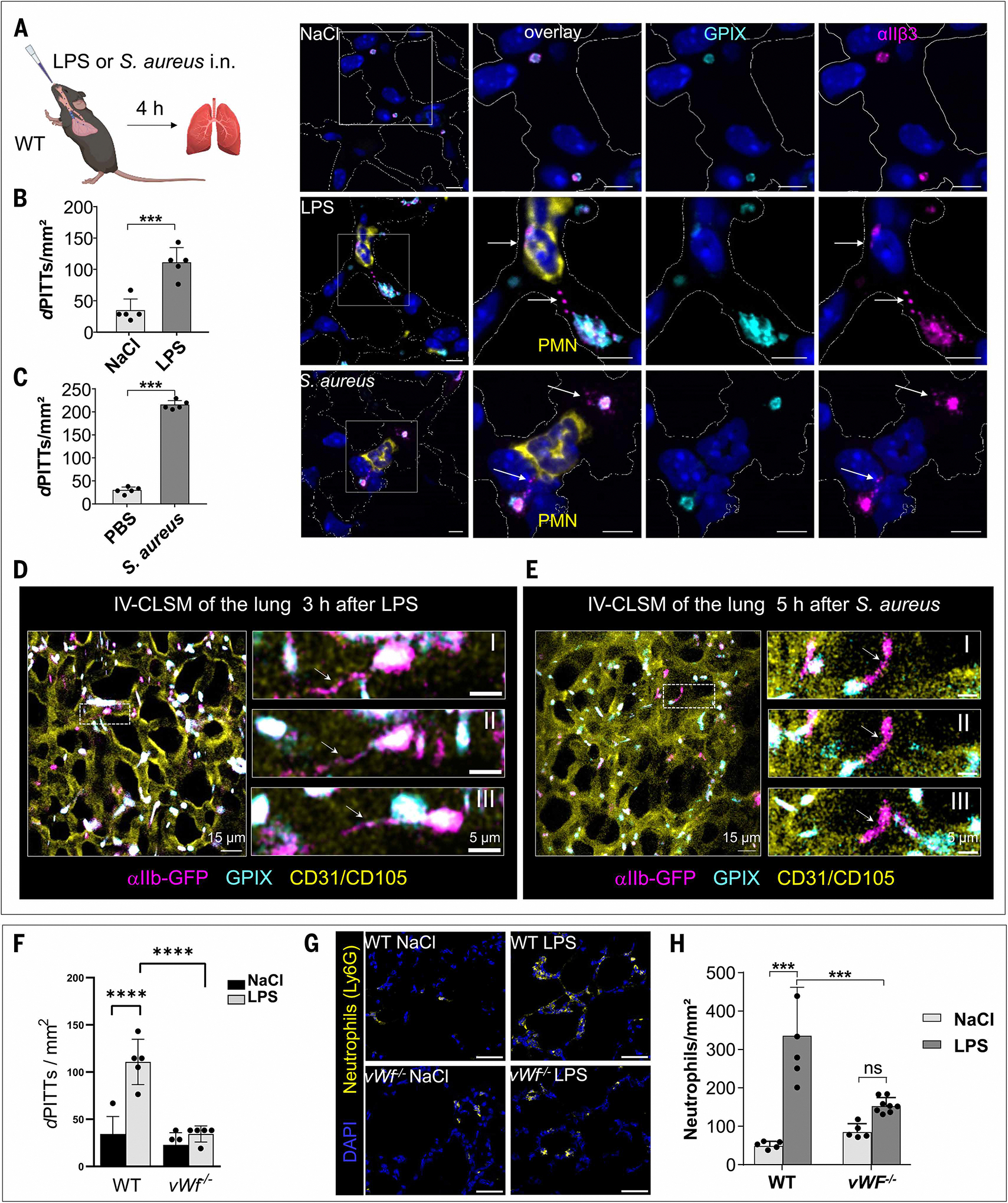
Platelets form and release PITTs under inflammatory conditions in mice. (**A** to **C**) WT mice were treated intranasally with NaCl (A), 10 μg/g BW LPS (B), or 1 × 10^6^ *S. aureus* (C). Lungs were harvested after 4 hours (LPS) or 6 hours (*S. aureus*), respectively. Cryosections were stained for platelet markers αIIbβ3 (magenta) and GPIX (cyan), or neutrophils (anti-Ly6G, yellow), and counterstained with DAPI (blue). The vessel bed (stained with anti-CD31) is highlighted by dashed lines. The vessel bed (stained with anti-CD105^AF647^) is highlighted by dashed yellow lines. *d*PITTs (αIIbβ3^+^/GPIX^−^, yellow arrows) were frequently observed in LPS-treated lungs but not in NaCl-treated controls. In *S. aureus*–infected lungs, PITTs were even more abundant, longer, and structurally more complex. Scale bar: 5 μm. Left: Quantification of *d*PITTs in LPS- (B) and *S. aureus*– (C) treated lungs versus NaCl-treated controls. Thirty fields (145 μm by 145 μm) per mouse were analyzed using maximum intensity z-projections. Data represent mean ± SD of five mice per group; ***P < 0.001 (Mann Whitney U test). PMN, polymorphonuclear leukocytes. (**D** and **E**) Representative intravital CLSM images showing PITT formation in the lungs of *Itga2b*^*GFP/GFP*^ mice after intranasal challenge with LPS (10 μg/g BW, 3 hours) or *S. aureus* (1 × 10^8^ CFU, 5 hours). Platelets were counterstained with anti-GPIX^AF546^ (5 μg, cyan), and the vasculature was labeled with anti-CD31^AF647^ and anti-CD105^AF647^ (10 μg each, yellow). The images are representative of five mice per group. (**F** to **H**) WT and *vWf*^*−/−*^ mice were treated intranasally with NaCl or 10 μg/g BW LPS and lungs were harvested after 4 hours. Cryosections were stained for αIIbβ3 (magenta), GPIX (platelets, cyan), or Ly6G (neutrophils, yellow) and counterstained with DAPI (blue). Quantification of *d*PITTs (αIIbβ3^+^/GPIX^−^) (F). Representative cryosection images showing neutrophil infiltration (anti-Ly6G, red) (G). LPS-induced neutrophil recruitment was significantly reduced in *vWF*^*−/−*^ mice compared to WT mice. Quantification of neutrophils/mm^2^ (H). Thirty fields (661.59 μm by 661.59 μm) per mouse were analyzed. In (B), (C), (F), and (H), individual data points show data from one mouse; bars are mean ± SD (*n* = 5 to 8 mice per group). Statistical analysis: one-way ANOVA with Tukeýs post-hoc test. *P ≤ 0.05; ***P < 0.001; ****P < 0.0001.

During thrombo-inflammation, platelets orchestrate immune cell trafficking and activation, as well as maintenance of vascular barrier function, and drive organ damage in diseases with limited treatment options (e.g., stroke, sepsis, or ARDS). Although αIIbβ3 plays a critical role in these pathologies (13–15), the underlying mechanisms remain poorly understood (48–50). We hypothesized that PITTs might contribute to thrombo-inflammatory cell recruitment and tissue injury. To test this, we used a model of lipopolysaccharide (LPS)–induced pulmonary inflammation (49). Mice received LPS (10 μg/g BW) or NaCl intranasally; after 4 hours, lungs were harvested and cryosections analyzed by CLSM. We double-stained for αIIbβ3 (JON6^AF647^) and GPIX (p0p6^AF488^ or p0p6^AF546^). Polarized and PITT-forming platelets were detected in LPS-treated, but not NaCl-treated, lungs (Fig. 4, A and B). The number of *d*PITTs also increased in LPS-treated lungs compared to control (Fig. 4B). The *d*PITTs were mainly attached to endothelial cells (ECs) and/or neutrophils. *d*PITTs were even more abundant in the lungs of mice intranasally infected with *Staphylococcus aureus* [1 × 10^6^ colony-forming units (CFUs) in 25 μl of phosphate-buffered saline (PBS); Fig. 4C]. Intravital microscopy in *Itga2b*^*GFP/GFP*^ mice confirmed PITT formation on ECs and neutrophils after LPS (Fig. 4D, fig. S8A, and movie S12) or *S. aureus* (Fig. 4E) challenge, though the small diameter (~50 to 200 nm) and the lung motion made visualization technically challenging. Robust PITT formation was also observed in the lungs of BALB/c mice infected with a mouse-adapted SARS-CoV-2 strain (rSARS2-N501Y_MA30_) (51, 52) (fig. S8B). These data suggested that PITT formation was a component of the thrombo-inflammatory response to pulmonary endotoxemia or infection.

At sites of inflammation, ECs release Weibel-Palade body contents and expose vWF multimers (53), a process that could explain platelet recruitment and PITT formation. We therefore subjected WT and *vWf*^*−/−*^ mice to LPS-induced lung inflammation. After 4 hours, lungs were isolated and cryosections analyzed by CLSM (49). WT controls showed a marked increase in *d*PITTs and neutrophil recruitment in response to LPS. By contrast, LPS treatment failed to induce the formation of *d*PITTs in *vWf*^*−/−*^ mice (Fig. 4F), and neutrophil infiltration was reduced by ~54% (Fig. 4G). Blockade of αIIbβ3 with JON/A-F(ab)_2_ [2 μg/g administered intravenously (i.v.)] had an even stronger anti-inflammatory effect, markedly reducing neutrophil recruitment, transmigration, NETosis, and tissue damage in this model (fig. S9), a process previously shown to occur independently of platelet aggregation (49).

**Fig. 5.**
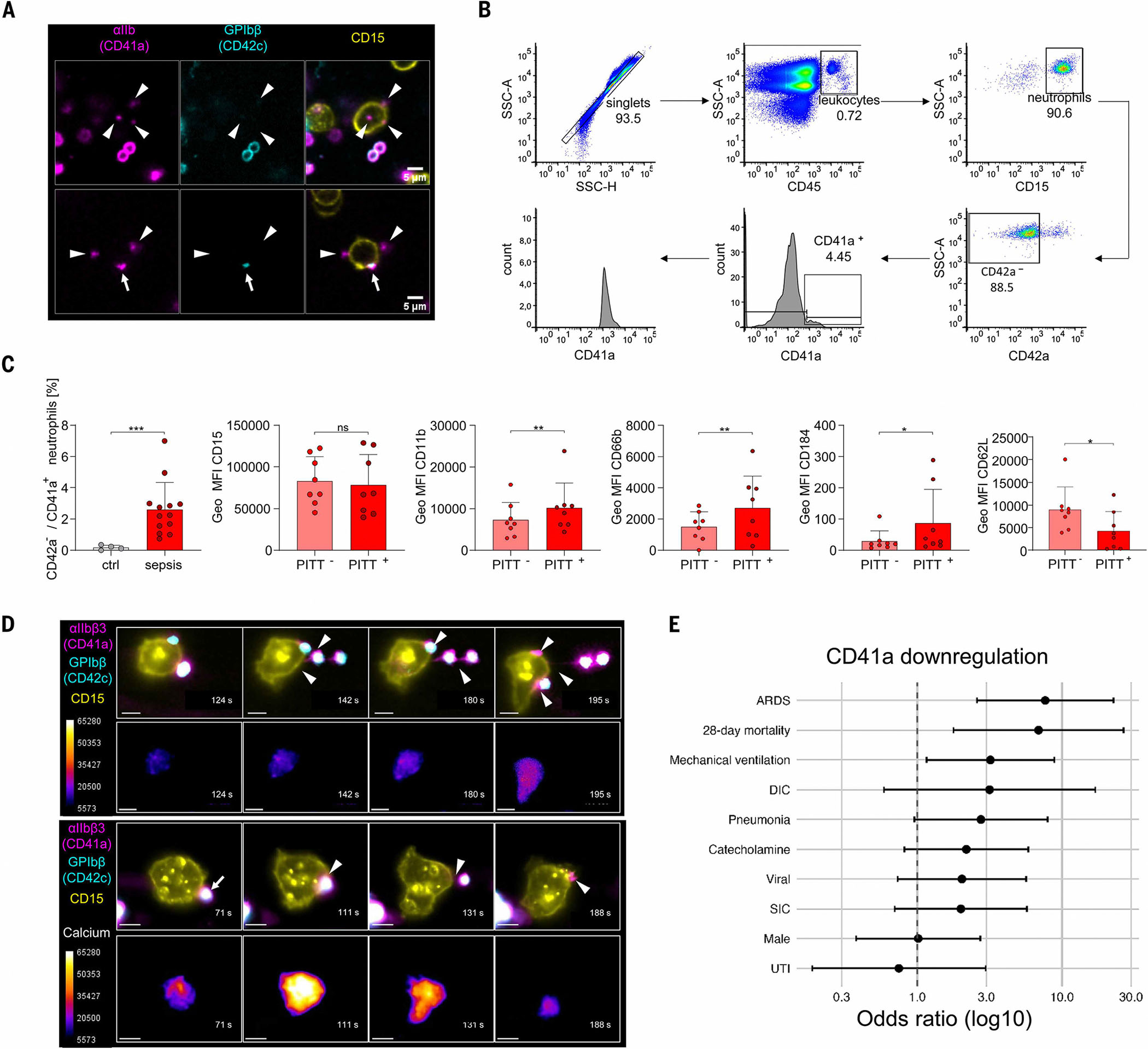
PITTs promote neutrophil activation and exacerbate inflammation in severely ill patients. (**A**) Confocal fluorescence microscopy of PITT-decorated neutrophils in the blood of a patient with severe sepsis. White arrowheads indicate PITTs (CD41a^+^/CD42c^−^). White arrows indicate CD41a/CD42c double-positive particles. Scale bar: 5 μm. (**B** and **C**) Flow cytometric analysis of PITT^+^ neutrophils in the blood of healthy controls and sepsis patients. (B) Gating strategy for identifying PITT^+^ neutrophils. (**C**) Frequency and activation phenotype of PITT^+^ versus PITT^−^ neutrophils. Data are represented as mean ± SD; each datapoint represents one individual (*n* = 8 per group). (**D**) Representative time-lapse images showing PITT formation and deposition on neutrophils in human blood. Neutrophils were isolated from healthy donors, stained for CD15 (yellow), loaded with the calcium indicator Fluo-4/AM (Fire LUT; scale bar in arbitrary units), and allowed to adhere to fibrin for 30 min at 37°C. Platelets from the same donor were double-labeled for GPIbβ (CD42c, cyan) and αIIbβ3 (CD41a, magenta), then perfused over the adherent neutrophils at a shear rate of 500 s^−1^. Scale bar: 5 μm. Time shown in seconds. The [Ca^2+^]_i_ increase in neutrophils upon PITT deposition is indicative of activation. (**E**) A patient cohort was stratified into CD41a^low^ and CD41a^high^ groups, with CD41a^low^ defined as the lowest quartile of expression. Odds ratios were calculated comparing CD41a^low^ to CD41a^high^. ARDS, acute respiratory distress syndrome; DIC, disseminated intravascular coagulation; UTI, urinary tract infection. Data are represented as mean ± SD (*n* = 8). Statistical testing: Mann-Whitney test (quantification of PITT^+^ neutrophils); Wilcoxon matched pairs signed rank test [comparison of PITT^+^ versus PITT^−^ neutrophils in (C)]. n.s. not significant; *P < 0.05; **P < 0.01; ***P < 0.001.

The proinflammatory function of αIIbβ3 and the detection of PITT-interacting neutrophils in inflamed mouse lungs led us to hypothesize that platelets may deposit PITTs directly onto neutrophils to modulate their function. Approximately 6% of neutrophils from patients with sepsis were decorated with PITTs (Fig. 5A). These PITT^+^ neutrophils were activated, as evidenced by the up-regulation of CD11b, CD66b, and CD184 expression and the down-regulation of CD62L, and were clearly distinguishable from PITT-negative (PITT^−^) neutrophils (Fig. 5, B and C). To monitor PITT formation on neutrophils in vitro, human neutrophils were allowed to adhere to fibrin, and whole blood, with platelets labeled with CD41a and CD42c, was perfused over them under flow. Frequent platelet–neutrophil interactions were observed, with clear deposition of PITTs on neutrophils (Fig. 5D and movies S13 and S14). These events triggered a discrete and transient increase in the concentration of intracellular Ca^2+^ ([Ca^2+^]_i_) in neutrophils (Fig. 5D and movies S13 and S14), indicating cellular activation. In samples of patients with sepsis, PITT formation was generally increased, with higher numbers of detached PITTs in blood smears compared to controls (fig. S10A), and was often accompanied by increased platelet-neutrophil complexes (PNCs) and microthrombi (fig. S10B). In some cases, platelets deposited numerous PITTs onto single neutrophils (fig. S10C), a phenomenon that was likewise observed in murine blood under corresponding conditions (fig. S10D and movie S15).

### The level of PITT formation is associated with disease severity

These results demonstrated that PITTs directly contribute to neutrophil activation, suggesting that platelets use this effector mechanism to promote inflammation. We next examined whether CD41a down-regulation— as a surrogate for PITT release—was associated with disease severity or clinical outcome in patient subcohorts. Reduced CD41a expression correlated with higher sequential organ failure assessment (SOFA) scores (correlation coefficient *r* = −0.39, *P* < 0.001), 28-day mortality (*r* = −0.27; *P* < 0.05), in-hospital stay (*r* = −0.27; *P* < 0.05), and the occurrence of ARDS (*r* = −0.40; *P* < 0.001). CD41a down-regulation also correlated negatively with the sepsis-induced coagulopathy (SIC) score (−0.40; *P* < 0.01) and the disseminated intravascular coagulation (DIC) score (−0.37; *P* < 0.01; tables S3 and S4). By contrast, there was no correlation with infection site or pathogen type, suggesting that PITT formation was linked to disease severity but not the underlying cause of the inflammation. On the basis of these findings, we hypothesized that low CD41a expression may identify patients at risk for adverse outcomes. Stratifying patients by platelet CD41a levels, we defined the lowest quartile as CD41a^low^. This subgroup showed markedly increased odds ratios (ORs) for 28-day mortality [OR = 6.9, confidence interval (CI) 1.8–26.6; *P* < 0.01], and ARDS (OR = 7.7, CI 2.6–22.7), linking increased PITT release to poor outcome and ARDS development (Fig. 5E).

## Discussion

Our findings provide evidence that during infection and inflammation, platelets shed part of their αIIbβ3/CD9 pool onto leukocytes and the activated endothelium via PITT formation—a process that does not involve classical platelet activation and amplifies the inflammatory response. In mice, αIIbβ3-depleted platelets retained normal life span, shape change, and degranulation capacity, but they exhibited defective adhesive function in thrombus formation and hemostasis. Bleeding and altered platelet function are hallmarks of sepsis and DIC (54, 55). Previous studies reported profound GPVI signaling impairment in critical illness (26), linked to reduced αIIbβ3 density and increased sensitivity to αIIbβ3 (GPIIb/IIIa) inhibitors under flow (24). Together, these findings suggest that PITT release not only drives inflammation but also may contribute to dysfunction associated with the “exhausted platelet phenotype” and bleeding risk in patients with severe thrombo-inflammatory disease (56).

PITT formation requires αIIbβ3 engagement by ligands or antibodies and the subsequent recruitment of nonengaged αIIbβ3 into the growing tether. This strongly implies that (i) ligated αIIbβ3 transduces signals that promote recruitment of distant αIIbβ3 heterodimers, and (ii) unoccupied αIIbβ3 exhibits high lateral mobility in the membrane. In adherent cells, proteins such as talin link the β-integrin cytoplasmic domains to the cytoskeleton, enabling force sensing, adhesion, and clustering of ligand-bound high-affinity integrins (1, 57, 58). In resting platelets, however, αIIbβ3 is not firmly anchored to the cytoskeleton—similar to quiescent monocytes, where only ~5% of the β2-integrin LFA-1 is cytoskeleton associated (59). High lateral mobility and the formation of microclusters upon ligand binding are thought to be crucial for stable shear-resistant adhesion of leukocytes (59, 60). However, studies in neutrophils have shown that the β2-integrin (Mac-1)–dependent formation of long membrane tethers promotes adhesion independently of outside-in signaling to the cytoskeleton (61). This supports the model in which cytoskeleton linkage is dispensable for integrin priming and intermediate-affinity adhesion, whereas high-affinity integrins predominantly engage ligands via cytoskeletal-dependent mechanisms (62, 63). These β2-integrin–dependent tethers likely represent precursors of elongated neutrophil-derived structures (ENDS), recently described as submicrometer particles formed during neutrophil rolling (64). This suggests that PITT formation may not be exclusive to platelets but may be a more generalized phenomenon in cell biology.

Like PITTs, ENDS are proinflammatory, arise during infection, and are released in conditions that do not favor firm integrin-mediated adhesion. These observations suggest that both structures extend the functional range of platelets and neutrophils, respectively, enabling spatially distant modulation of inflammation. The high density of αIIbβ3 in PITTs—apparently in low-or intermediate-affinity state—points to a direct proinflammatory role of the integrin that differs from its canonical function. One possible mechanism could be integration of αIIbβ3 into the plasma membrane of the target cell. Indeed, it has been reported that neutrophils can acquire αIIbβ3 from platelet-derived microparticles, which then colocalize with β2-integrins and enhance nuclear factor κB (NF-κB) activation (65). In addition, PITTs likely modulate the function of target cells by transferring other membrane proteins and cytoplasmic cargo whose composition may vary considerably owing to changes in the transcriptomic profile of megakaryocytes and platelets in inflammatory disease settings (66).

In conclusion, we have identified a mechanism by which platelets separate their major integrin, αIIbβ3, into a distinct proinflammatory organelle, the PITT. We found that the (partially) integrin-depleted platelets stay viable, remain in a resting state, and are not cleared from the circulation, but show reduced capacity to aggregate and form thrombi. This highly organized separation of the plasma membrane integrin pool explains the loss of αIIbβ3 in patients with COVID-19 or other severe inflammation. We suspect that excessive PITT formation contributes to the bleeding tendency combined with microvascular inflammation observed in patients with severe infection, sepsis, or related pathologies.

## Materials and methods

### Human material

Blood samples from healthy volunteers were collected after written informed consent. Only adults were recruited as healthy controls. According to our study protocol, the only parameters recorded were age (median 26 years, interquartile range 23–30) and sex (50.9% male), and there is no information on body mass index or ethnic background. Adult patients with infection, sepsis, or COVID-19 were recruited between April 2020 and March 2023 at the University Hospital Würzburg after informed consent was given by the patient or legal guardian. SARS-CoV-2 infection was confirmed by polymerase chain reaction testing. Sepsis was defined according to the Sepsis-3 criteria due to an increase of the SOFA score of 2 points (67). Non–SARS-CoV-2 infection patients were stratified to the “infection” cohort according to a SOFA score of 0 or 1 point. Blood withdrawal was scheduled at the day of hospital/ICU admission. Patients were excluded if they had aplasia, were pregnant, or were receiving ECMO-based treatment. The study was conducted in accordance with the Declaration of Helsinki and approved by the Institutional Review Boards (IRBs) of the University of Würzburg (EV 94/19 and COVID-19 amendment). Age and sex of all study participants are reported in table S1. As outlined above, no information on ethnic background was documented. Based on the location of our hospital (UKW, Germany), we can readily state that all or the vast majority of our study participants are of white/Caucasian background.

#### Animals:

All animal experiments were performed in accordance with the National Institutes of Health Guidelines for the Care and Use of Laboratory Animals, in compliance with the ARRIVE guidelines (68), and approved by the local authorities (District Government of Lower Franconia, Germany). Mice of both sexes, aged 4–19 weeks, were maintained under specific pathogen-free conditions at the University Hospital Würzburg. Animals were housed in open cages (3–8 per cage depending on body size) with softwood bedding, nesting material, food and water ad libitum, under a 12/12 hours light/dark cycle at 20°–24°C and 45–65% humidity. Only healthy animals were included in experiments; no additional inclusion or exclusion criteria were applied. Euthanasia was performed by cervical dislocation. Treatment groups were assigned using randomizer.org, and surgery as well as analysis were carried out in a blinded fashion. Sample sizes were determined prior to initiation of the study as part of the animal experiment application. Power calculations were performed with G*Power software based on effect sizes observed in comparable previously published experiments. The resulting group sizes ensured adequate statistical power while minimizing animal use in accordance with the 3R principles. C57BL/6JRj mice maintained under specific pathogen–free conditions were used as wild-type (WT) or control (Ctrl) mice. Constitutive KO mice for von Willebrand factor (*vWF*^−/−^) (69), FcγRIIB (*Fcgr2b*^*−/−*^) (70), and FcγRIII (*Fcgr3*^*−/−*^) (71) were described earlier and backcrossed onto the C57Bl/6J background for at least 10 generations. *Arf6*^*fl/fl*^ mice (Jax order no. 028669) were intercrossed with PF4-Cre mice (72) to generate mice lacking Arf6 in megakaryocytes and platelets (*Arf6*^*fl/fl Pf4-cre*^). *Fcgr3:Fcgr2b*–double knockout (DKO) mice were generated by intercrossing *Fcgr2b*^*−/−*^ and *Fcgr3*^*−/−*^ mice (see table S5 for all mouse strains used in this study). Animal experiments were approved by the district government of Lower Franconia (Regierung von Unterfranken). Itga2b-GFP mice were generated by inserting the eGFP coding sequence into exon 30 of Itga2b using CRISPR/Cas9, based on a prior study expressing GFP-tagged human αIIb in CHO cells (29). BALB/c mice (8–10 weeks old) were obtained from Charles River Laboratories and were housed under specific pathogen–free conditions.

#### Blood smears:

Peripheral blood was collected and immediately smeared onto clean glass slides, followed by air-drying for 30 min. Dried smears were stained with May-Grünwald solution (Merck) for 5 min, then with Giemsa solution (Merck) for 15 min. Alternatively, smears were fixed with glyoxal and stained for 45 min each with fluorescently labeled antibodies targeting αIIbβ3, CD9, and indicated subunits of the GPIb-IX-V complex (see table S6 for all primary antibodies used in this study). Excess stain was removed by rinsing with 0.1% Tween-20 in PBS, followed by air drying. Imaging was performed on a Leica TCS SP8 inverted confocal microscope using a 63x oil immersion objective (NA 1.4) to assess platelet morphology and tether formation.

#### Platelet preparation:

Mouse blood was collected into heparin (20 U/ml, Ratiopharm) and platelet-rich plasma (PRP) was obtained by centrifugation at 300*g* for 5 min at room temperature (RT). For the preparation of washed platelets, PRP was centrifuged at 640*g* for 5 min at RT. The platelet pellet was resuspended in modified Tyrode-HEPES buffer [134 mM NaCl, 0.34 mM Na_2_HPO_4_, 2.9 mM KCl, 12 mM NaHCO_3_, 5 mM HEPES, 1 mM MgCl_2_, 5 mM glucose, and 0.35% bovine serum albumin (BSA); pH 7.4)] in the presence of prostacyclin (0.5 μM) and apyrase (0.02 U/ml). Platelets were finally resuspended in the same buffer without prostacyclin (pH 7.4) but with 0.02 U/ml apyrase and incubated at 37°C for 30 min before use.

For the preparation of human platelets, whole blood of healthy volunteers was taken in 1/10 volume of acid-citrate-dextrose, centrifuged at 200*g* for 10 min. Apyrase (0.2 U/ml) and PGI_2_ (0.1 μg/ml) were added to the PRP, and the platelets sedimented at 800*g* for 10 min., washed twice with Tyrode’s buffer without Ca^2+^ in the presence of apyrase and PGI_2_, and then allowed to rest at 37°C for 30 min before use.

#### Transmission electron microscopy (TEM):

Washed platelets in a concentration of 3 × 10^5^ platelets/μl were fixed with 2.5% glutaraldehyde (Agar Scientific) in cacodylate buffer (pH 7.2, AppliChem). Epon 812 (Serva) was used to embed platelets (73). After generation of ultrathin sections, platelets were stained with 2% uranyl acetate (Electron Microscopy Science) and lead citrate (Merck). Sections were analyzed on a JEM 1400 (JEOL) electron microscope. For TEM of the liver, perfusion-fixed liver was incubated overnight at 4°C in 2.5% glutaraldehyde (Agar Scientific) in cacodylate buffer (pH 7.2, AppliChem) and then processed as described for platelets.

#### Platinum replica electron microscopy:

Platelet cytoskeleton was analyzed as described previously (73–75). In brief, washed platelets were spun onto poly-L-lysine–coated coverslips and fixed in 0.05% glutaraldehyde and 0.04% paraformaldehyde (PFA). Finally, all samples were sequentially incubated with 1% glutaraldehyde, 0.1% tannic acid, and 0.2% uranyl acetate. Dehydration was performed by transferring samples through graded acetone. Critical point drying was done in a Leica EM CPD300. Samples were finally coated with 1.2 nm of platinum with rotation at 45°C and 3 nm of carbon at 90°C without rotation under high vacuum in a Leica EM ACE600. Replicas were floated, picked up on formvar-carbon–coated grids, and examined with a JEOL JEM-2100.

#### Scanning electron microscopy (SEM):

For scanning electron microscopy, washed platelets (250.000/μl) were stimulated with the respective agonist for 15 min. Samples were fixed with 5% glutaraldehyde in 100 mM cacodylate buffer for 15 min at 37°C and for 1 hour at RT after layering on poly-L-lysine–coated cover slips followed by incubation at 4°C overnight. The samples were washed five times with 100 mM phosphate buffer (100 mM KH_2_PO_4_/100 mM Na_2_HPO_4_, volumes 12.5/87.5) and further applied to a 30–100% acetone gradient for dehydration and stored in 100% acetone overnight. Critical point drying was performed in a Leica EM CPD300. Samples were finally coated with gold in an Emitech sc7320 sputter coater and visualized with a JEOL JSM 7500F scanning electron microscope.

For micropatterns, samples were fixed with 6.25% glutaraldehyde in 50 mM phosphate buffer (pH 7.4) overnight at 4°C. The samples were washed five times with 100 mM phosphate buffer (100 mM KH_2_PO_4_/100 mM Na_2_HPO_4_, volumes 12.5/87.5) and further applied to a 30–100% acetone gradient for dehydration and stored in 100% acetone overnight. Critical point drying was performed in a Leica EM CPD300. Samples were finally coated with gold in an Emitech sc7320 sputter coater and visualized with a JEOL JSM 7500F scanning electron microscope.

#### *d*STORM microscopy:

Single-color *d*STORM imaging was performed as previously described (31), using a widefield setup based on an inverted microscope (Olympus IX-71) equipped with a 60x oil immersion objective (NA 1.45; Olympus) and a diode laser (641 nm; Cube 640–100C, Coherent) delivering 2–10 kW/cm^2^ irradiation. Emission light was separated from excitation using a dichroic mirror (ZT 405/514/635rpc, Chroma Technology Corp.) and a bandpass filter (Em01-R442/514/647–25; Semrock), and projected onto an EMCCD camera (iXon DU-0897, Andor). Images were acquired at 20-ms exposure over 15,000 frames in a photoswitching buffer (pH 7.4) containing 100 mM β-mercaptoethylamine, without oxygen scavenger.

Dual-color *d*STORM imaging was performed on the same optical setup. Alexa-Fluor^647^, Alexa-Fluor^532^, or CF^®^568 were excited using respective diode lasers (Genesis MX639 and MX514, Coherent) at 2–10 kW/cm^2^. Light was separated using a dichroic mirror (ZT 405/514/635rpc, Chroma) and a bandpass filter (Em01-R442/514/647–25, Semrock), followed by a dichroic beam splitter and emission filters (Brightline HC 679/41 and HC 582/75; Semrock). Images were projected onto two EMCCD cameras (iXon Ultra DU-897, Andor) and recorded at 20-ms exposure over 15,000 frames (red) or 30,000 frames (green) in photoswitching buffer (pH 6.9, 100 mM β-mercaptoethylamine, no oxygen scavenger).

To correct chromatic aberration, TetraSpeck fluorescent beads were imaged in both channels across the region of interest and chromatic shift correction was applied using the bUnwarpJ plugin in Fiji (76).

Total internal reflection fluorescence (TIRF) illumination was used for imaging spread or pattern-adherent platelets; epifluorescence (EPI) was used for resting platelets. Super-resolution image reconstruction was performed using rapidSTORM 3.3 (open-source).

#### Confocal microscopy of platelets:

Washed platelets were seeded onto 2 M glycine coated 8-well chamber slides (8 Chambered Cover Glass, #1.5, Cellvis) for 15 min., incubated with AlexaFluor^647^ labeled MWReg30 or other indicated antibodies for 15 min. before fixation with 3% glyoxal solution (77). For cytoskeletal staining washed platelets were seeded onto 2 M glycine-coated wells for 15 min. and incubated with 10 μg/ml unlabeled MWReg30 for 15 min, then fixed with cytoskeleton buffer and stained with phalloidin-AlexaFluor^−488^ (Sigma-Aldrich) and anti-α-tubulin antibody (clone B-5–1-2, Sigma-Aldrich) labeled with AlexaFluor^647^ (74). Imaging was performed using a confocal laser-scanning microscope (TCS SP5 or TCS SP8, Leica Microsystems, Wetzlar, Germany) equipped with CFI Plan Apochromat VC lenses (NA 0.75, 1.25 and 1.4) at magnifications of 200x, 400x or 600x. Images were acquired using NIS Elements AR or LAS X software with settings adjusted according to the Nyquist sampling criterion and acquisition frequencies of 100–200 Hz.

For staining of human platelets, PRP was isolated from citrated whole blood. For the 15 min. time point, platelets were incubated with anti-GPIbβ^AF647^ (clone p0p1) together with 10μg/ml of anti-αIIbβ3^AF647^ (clone Hip8) or anti-αIIbβ3^AF546^ (clone Gi5); platelets were then fixed using 4%PFA/0.75% glutaraldehyde for 15 min. at RT. For the t0, platelets were instead fixed before staining with the above-described antibodies. Finally, platelets were deposited on poli-L-lysine–coated 8-well chamber slides (8 Chambered Cover Glass, #1.5, Cellvis) for 15 min at RT and imaged by confocal microscopy.

#### Confocal microscopy of platelet-neutrophil interaction:

Citrate anti-coagulated whole blood was diluted 1:10 in HEPES-Tyrode’s buffer and fixed with 4% PFA for 20 min at room temperature. Fixation was stopped with 700 μl HEPES-Tyrode’s buffer followed by centrifugation for 10 min at 500*g* and resuspension of cells in HEPES-Tyrode’s buffer. Neutrophils were stained using an APC-conjugated anti-CD15 antibody (clone HIM1, BioLegend), platelets were labeled with an Alexa-Fluor^405^-conjugated anti-GPIbβ antibody (clone p0p1), and a fluorescein isothiocyanate (FITC)–conjugated antibody raised against αIIbβ3 (clone HIP8, BD Pharmigen) for 15 min at room temperature in the dark. Cells were washed in HEPES-Tyrode’s buffer, centrifuged for 10 min at 500*g* and resuspended in 500 μl HEPES-Tyrode’s buffer. Stained cells were finally pipetted onto μ-Slide 8 well glass bottom Ibidi-chambers (Ibidi). Image acquisition was done using a Leica TCS SP8 inverted confocal microscope with a 40x/NA 1.3 oil objective. Image processing was performed with Image J (Version 2.0.0-rc-43/1.51 g, NIH, MD, USA) software.

#### Immunoblotting:

Proteins from washed platelet lysates were separated by sodium dodecyl sulfate–polyacrylamide gel electrophoresis (SDS-PAGE) using 4–12% Bis-Tris gradient gels (NP0335, Invitrogen) and transferred to polyvinylidene difluoride (PVDF) membranes as described previously (78). After blocking in 5% non-fat milk in TBS-T, membranes were incubated overnight at 4 °C with the following primary antibodies: anti-β-actin (A2066, Sigma-Aldrich), anti-GAPDH (glyceraldehyde-3-phosphate dehydrogenase) (G9545, Sigma-Aldrich), anti-CD41 (sc-15328, Santa Cruz Biotechnology), anti-β3 (clone EDL1) or anti-FcγRIIb (96397, Cell Signaling).

For detection, membranes were incubated for 1 hour at room temperature with horseradish peroxidase (HRP)–conjugated secondary antibodies or directly with an HRP-conjugated anti-β3 antibody (clone EDL1, in-house generated). Signal detection was performed using enhanced chemiluminescence solution (MoBiTec), and images were acquired using an Amersham Imager 600 (GE Healthcare).

#### Flow cytometry:

To assess platelet size and surface receptor expression, heparinized mouse blood was diluted 1:20 in HEPES-Tyrode’s buffer and stained for 15 min with saturating concentrations of fluorophore-conjugated antibodies. Samples were immediately analyzed on a FACSCalibur or FACSCelesta flow cytometer (both Becton Dickinson) as described previously (79). For activation studies, washed platelets were resuspended in calcified Tyrode’s-HEPES buffer (2 mM Ca^2+^) after washing twice with Tyrode’s-H EPES buffer. The washed murine platelets were incubated with each agonist as indicated in the presence of saturating concentrations of fluorophore conjugated antibodies against activated αIIbβ3 (JON/A^PE^, Emfret Analytics) and P-selectin (WUG.E9^FITC^, Emfret Analytics) for 6 min at 37°C followed by 6 min at room temperature. At the end of the incubation period, 500 μl PBS were added and samples were analyzed.

For surface marker profiling of human platelets, blood was collected into citrate-anticoagulated tubes (Sarstedt, 3.2%). After dilution 1:10 in HEPES-Tyrodes buffer (26), platelets were labeled with antibodies against αIIbβ3 (CD41a-APC (HIP8)), CD42a-PerCP (Beb1) and CD9-FITC (M-L13) (all Becton Dickinson). Leukocytes / neutrophils were stained with antibodies against CD15-FITC (HIM1), CD11b-PE (ICRF44), CD184-APC-Cy7 (FN50) (all Becton Dickinson), CD66b.PerCP/Cy5.5 (G10F5) and CD62L-BV785 (DREG-56; both BioLegend) for 15 min prior to stopping with FACS buffer. Analysis was performed using a FACSCelesta flow cytometer (Becton Dickinson) with 10,000 events recorded within the FSC/SSC defined platelet gate. Data were analyzed using FlowJo Vers.10 (Becton Dickinson). Surface expression levels were depicted as Geo-MFI values and compared to a reference cohort of healthy donors including a daily control sample.

#### Aggregometry:

Washed platelets (160 μl with 0.5×10^6^ platelets/μl) were stimulated in the presence (10 μg/ml collagen and 10 μg/ml convulxin) or absence (0.01 u/ml thrombin) of 70 μg/ml human fibrinogen (Sigma). For ADP-induced aggregation, platelet-rich plasma (PRP) was used and stimulated with 10 μM ADP. Light transmission was recorded on a four-channel aggregometer (Fibrintimer, APACT, Hamburg, Germany) for 10 min and expressed in arbitrary units, with buffer or platelet poor plasma representing a light transmission of 100% (80).

#### Bleeding time:

Mice were anesthetized and a 1 mm segment of the tail tip was removed with a scalpel (81). Tail bleeding was monitored by gently absorbing blood with filter paper at 20-s intervals without making contact with the wound site. Cessation of bleeding was defined as the absence of blood on the filter paper.

#### Intravital microscopy of thrombus formation in FeCl_3_-injured mesenteric arterioles:

Four- to five-week-old mice were anesthetized, and the mesentery was exteriorized through a midline abdominal incision as described before (79). Arterioles were visualized using a Zeiss Axiovert 200 inverted microscope equipped with a 10×/0.3 NA objective, a 100 W HBO fluorescence light source, and a CoolSNAP-EZ camera (Visitron). Digital images were recorded and analyzed offline using MetaVue software. Injury was induced by topical application of a 3 mm filter paper saturated with 20% FeCl_3_. Adhesion and aggregation of fluorescent labeled platelets (DyLight-488-conjugated anti-GPIX IgG-derivative) in arterioles were monitored for 40 min or until complete occlusion occurred (blood flow stopped for longer than 1 min).

#### Platelet adhesion under flow:

Micropatterned coverslips with rectangular 40 × 2 μm areas were generated using a PRIMO maskless UV-patterning system (Alvéole, Paris, France) and coated with vWF, fibrin, or MWReg30 (5 μg/ml each). For the formation of fibrin on micropatterns, 15 μl of fibrinogen solution (100 μg/ml) was applied and incubated for 30 min at 37°C. Following a blocking step with 1% bovine serum albumin (BSA) for 1 hour, thrombin (0.1 U/ml) was added and the incubation continued for another 30 min at 37°C.

For coating with vWF, the micropatterns were first incubated with anti-vWF antibodies overnight at 4°C. Nonspecific binding sites were then blocked with 1% BSA for 1 hour. The micropatterns were then incubated with mouse plasma for 3 hours at 37°C. Heparinized whole mouse blood—obtained from either wild-type (C57Bl/6J) or Itga2b-GFP reporter mice—was diluted in a ratio of 2:1 in Tyrode’s-HEPES buffer containing Ca^2+^ comparable to flow adhesion on collagen (73). Where indicated, platelet activation was inhibited by preincubation with either 0.1 μg/ml PGI_2_/300 μM ASA (acetylsalicylic acid, Bayer AG)/0.02 U/ml apyrase, or 20 μM BAPTA combined with 500 μM EGTA. Whole blood was incubated for 5 min at 37°C with either anti-αIIbβ3^AF647^ (except for reporter mice) combined with an anti-GPIX^DyLight488^, or an anti-CD9^AF647^ combined with anti-αIIbβ3^AF532/568^, depending on the coating used on the patterns. The PDMS stencils were removed from the coverslips, which were then placed into the middle of the flow chamber setup to align with the 50 μm channel. The blood was diluted 2:1 with Tyrode’s buffer without Ca^2+^ perfused over the patterns at 1000 s^−1^ for 5 min while imaged in a Leica DMI6000B inverted microscope (63x/1.3 glycerol HCX PL APO objective) using a Leica DFC 360 FC camera. After the run, the slides were washed with Tyrode’s buffer without Ca^2+^ for 5 min at 1000 s^−1^.

#### Platelet-neutrophil interaction under flow:

Coverslips were incubated with 100 μg/ml fibrinogen in PBS for 30 min at 37°C. Subsequently, 0.3 U/ml thrombin was added for 30 min at 37°C.

Mouse neutrophils were isolated from femoral and tibial bone marrow using Histopaque density gradient centrifugation. Separation was performed with Histopaque-1119 (11191, Sigma) and Histopaque-1077 (10771, Sigma), and all steps were carried out at low temperature to minimize neutrophil activation.

Human neutrophils were isolated from peripheral blood of healthy donors via venipuncture (9 ml tubes, 3.2% trisodium citrate). After centrifugation at 150*g* for 20 min, the buffy coat was collected, and residual red blood cells were lysed using ACK buffer (0.15 M NH_4_Cl; 0.01 M KHCO_3_; 0.1 mM EDTA). Finally, the cells were centrifuged at 400 g for 10 min and resuspended in Neutrophil isolation buffer (CaCl_2_ 1 mM, MgCl_2_ 0.5 mM, HEPES 10 mM, BSA 0.25%, Glucose 10 mM in PBS).

Neutrophils were stained with anti-Ly6G^AF647^ (mouse) or anti-CD15^APC^ (human), loaded with Fluo-4/AM (F14201, Thermo Fisher) and then allowed to adhere for 30 min at 37°C on a fibrin-coated surface. Anticoagulated mouse blood was stained for 5 min with anti-αIIbβ3^Fab-AF546^ and anti-GPIX^AF405^, human blood from healthy volunteers was stained for 5 min with anti-αIIbβ3^AF546^ and anti-GPIbβ [p0p1^AF405^ (82)]. Labeled blood was then perfused over the adherent neutrophils at the indicated shear rate. Videos and images were acquired using a Thunder Imager DMi8 microscope (63x/1.3 glycerol HCX PL APO objective).

#### IHC staining of liver cryosections:

For immunohistochemical staining of liver cryosections (47), tissue samples were embedded in Tissue-Tek O.C.T. compound (Sakura Finetek) and snap-frozen in liquid nitrogen. Sections of 7 μm thickness were prepared and fixed in 4% paraformaldehyde. Endogenous peroxidase activity was blocked with hydrogen peroxide (H_2_O_2_), and non-specific binding was blocked using 3% BSA in PBS. For platelet staining, sections were incubated with HRP-conjugated anti-GPIbβ antibodies (Emfret Analytics). Signal detection was performed using 3-Amino-9-ethylcarbazole (AEC), followed by hematoxylin counterstaining. Slides were imaged using a Leica Thunder Imager DMi8 equipped with an Andor Zyla 4.2 brightfield camera and analyzed using LAS X software (version 3.7), with 10× or 20× objectives.

For immunofluorescence microscopy, vasculature was visualized by intravenous injection of anti-CD105 AlexaFluor^647^ (clone MJ7/19, purified in-house, 0.4 μg/g body weight). Washed platelets (prepared as described above) were incubated for 10 min with 5 μg/ml AlexaFluor^546^-labeled anti-GPIX mAb (44) and 5 μg/ml AlexaFluor^488^-labeled MWReg30, then washed to remove unbound antibody. A total of 1.5 × 10^8^ platelets (in 150 μl Tyrode’s buffer) were transfused intravenously. Liver tissue was fixed in 4% paraformaldehyde overnight, embedded in Tissue-Tek, frozen, and sectioned at 7 μm. Confocal imaging was performed using an inverted Leica SP8 microscope equipped with 63x oil immersion objective.

#### Determination of body temperature:

To assess hypothermia, body temperature was measured at the indicated time points using a rectal probe following intravenous injection of vehicle or MWReg30 (3 μg/g body weight).

#### Intravital confocal laser-scanning microscopy (IV-LSM) of the liver:

Intravital imaging was performed using a Leica TCS SP8 inverted confocal microscope equipped with a Cube Unit to maintain a temperature-controlled environment for anesthetized mice. Vasculature was visualized by intravenous injection of anti-CD105-AlexaFluor^647^ (clone MJ7/19, purified in-house, 0.4 μg/g body weight). Mice were anesthetized with 0.5 mg/kg Medetomidin (Pfizer), 5 mg/kg Midazolam (Roche) und 0.05 mg/kg Fentanyl (Janssen-Cilag GmbH). After shaving and disinfecting the surgical area, a midline abdominal incision was performed. Superficial vessels in the skin and peritoneal muscle were cauterized before removal. The connecting ligaments were carefully severed to expose the left medial liver lobe. To prevent desiccation and minimize motion artifacts caused by respiration, the liver lobe was covered with a Kimwipe moistened with warm saline and gently stabilized.

Imaging was performed with a 25x water immersion objective on the Leica TCS SP8 microscope. Mice received either MWReg30^AF488^ (0.2 μg/g BW) or pre-labeled platelets. Washed platelets (prepared as described above) were incubated for 10 min with 5 μg/ml anti-GPIX^AF546^ (44) and 5 μg/ml MWReg30^AF488^, washed again to remove unbound antibody and 1.5 × 10^8^ platelets (in 150 μl Tyrode’s buffer) were transfused into the anesthetized mice.

Time-lapse image stacks were processed using Huygens Professional (version 21.04.0p1, Scientific Volume Imaging B.V., Hilversum, The Netherlands). Deconvolution and temporal stabilization were performed using cross-correlation and Lanczos interpolation; full cropping was applied. Frames exhibiting motion artifacts or excessive photobleaching were removed. Bleaching correction was performed using the Huygens Bleaching Corrector. Resulting files were converted to Imaris Classic format and segmented in Imaris (version 9.7, Bitplane AG, Zurich, Switzerland). The blood vessel surface was reconstructed using the Surface Model tool based on anti-CD105 fluorescence, with smoothing set to 500–700 nm (5–7 pixels) and manually adjusted absolute threshold values. Integrin and CD105 channels were masked using the reconstructed CD105 surface. Videos were rendered using the Imaris recorder at 40 frames per second.

For visualization, Fiji (ImageJ version 1.53c) (83) was used to generate maximum intensity z-projections. These were subsequently imported into Imaris for segmentation and surface rendering as described above. Final images were exported using the Imaris Snapshot tool.

#### Lung injury:

Mice were anesthetized with 1.5% isoflurane in O_2_ and placed in a 45° inclined supine position. Lipopolysaccharide (LPS, *E. coli* O111:B4, Sigma) or *Staphylococcus aureus* (strain HG001) was administered intranasally at a dose of 10 μg/g body weight (BW) or 1 × 10^6^ CFU in PBS, respectively. Control animals received 0.9% NaCl. After 3–5 hours, mice were terminally anesthetized with ketamine/xylazine and exsanguinated via transection of the inferior vena cava. The trachea was cannulated, and lungs were inflated with 0.8 ml of a 1:1 mixture of optimum cutting temperature (O.C.T^™^) compound (Tissue-Tek^®^, Sakura) and 10% sucrose in PBS. The trachea was ligated, and the lungs were excised en bloc, followed by heart removal. Lungs were embedded in Tissue-Tek^®^ cryomolds, overlaid with O.C.T. compound, and snap-frozen in liquid nitrogen. Cryosections of 7 μm thickness were prepared using a LEICA CM1950 cryostat and mounted on Superfrost Plus slides (Thermo Scientific). Sections were fixed in glyoxal, and non-specific binding was blocked with 5% BSA (w/v) and 3% goat serum (v/v) in PBS. For platelet staining, sections were probed with 5 μg/ml MWReg30^AF647^ and fluorescently labeled anti-GPIX derivatives (home-made) as indicated in the figure legends. Vessels were stained using 10 μg/ml fluorescently labeled anti-CD31 antibody (in-house generated). For neutrophil staining, sections were probed with 2 μg/ml fluorescently labeled anti-Ly-6G antibody (BioLegend). For detection of neutrophil extracellular traps (NETs), cryosections were incubated with rabbit anti-mouse histone H3 (citrullinated R2+R8+R17; Abcam) diluted in blocking buffer for 1 hour at RT. After three washing steps, sections were incubated with goat anti-rabbit IgG^AF546^ (1:300, Invitrogen) diluted in dilution buffer for 45 min at RT. Sections were analyzed using a Leica TC SP8 inverted confocal microscope. Images were preprocessed using Huygens Deconvolution software (Huygens Professional 20.10.0p1 64bit, Scientific Volume Imaging B.V., Hilversum, The Netherlands) to obtain higher contrast and better signal-to-noise ratio (SNR). Brightness and contrast were optimized, and analysis was performed using ImageJ software (83).

Intravital confocal microscopy of ventilated mouse lung was performed as previously described (49). After 3–5 hours of LPS treatment or *S. aureus* infection, mice were anesthetized by intraperitoneal injection of ketamine (100 mg/kg body weight) and xylazine (16 mg/kg body weight). A cocktail of antibodies, consisting of 7 μg anti-mouse CD31^AF647^ (in-house generated), 5 μg anti-mouse Ly6G^AF488^ (BioLegend), and 5 μg anti-mouse GPIX^AF546^ (in-house generated), was administered intravenously in a bolus of 100 μl. The mice were tracheotomized and ventilated at a rate of 150 strokes per minute, with a tidal volume of 10 μl entrained oxygen (100%) per gram body weight. After ventilation, the mice were placed in the right lateral decubitus position, and a thoracotomy was performed to expose the left lung lobe. A custom-made thoracic suction window was used to immobilize the lung surface, applying a negative pressure of ~30 cm H_2_O. Intravital microscopy was conducted using a Leica TCS SP8 confocal laser-scanning microscope with a 25x water immersion objective (HC FLUOTAR L 25x/0.95, Leica Microsystems), 8000 Hz resonant scanner, digital zoom of 2, and a resolution of 1024 × 1024 pixels. Images were acquired every second for 5 min, with a total of 6 fields of view (FOV) captured over 60 min.

#### Mouse SARS-CoV-2 model:

The mouse-adapted SARS-CoV-2 strain rSARS-CoV-2-N501YMA30 (51) was propagated in Vero E6-TMPRSS2 cells for 48–73 hours and titrated by plaque assay under biosafety level 3 (BSL-3) conditions. For aerosol infection, non-anesthetized mice were placed in nose-only Allay restrainers connected to an inhalation chamber (DSI Buxco Respiratory Solutions), as previously described (52, 84). Mice were exposed for 50–60 min to aerosolized virus to deliver a target accumulated inhaled dose of 1 × 105 TCID50. Exposure time varied according to the virus dilution and number of mice per session. Primary airflow and chamber pressure were maintained at 0.5 liter/min/port and −0.5 cmH_2_O, respectively. Control mice received aerosolized PBS (125 μl/mouse). Infected animals were monitored daily for weight loss, clinical symptoms, and respiratory function. Infectious experiments were performed in designated BSL-3 containment facilities.

#### Data analysis:

Unless otherwise stated, data are presented as mean ± standard deviation (SD) from at least two independent experiments per group. Normality of data distribution and equality of variances were assessed prior to statistical testing, and the appropriate test was selected accordingly. Comparisons involving multiple groups were corrected using the Holm–Šidák method. For our clinical cohorts, correlation analyses were performed using the Pearson and Spearman correlation method. Correction for multiple testing was performed using the Holm-Bonferoni method. Associations between low platelet CD41a expression (defined as the lowest quartile) and binary clinical outcomes were assessed using chi-square tests. Odds ratios with 95% confidence intervals were calculated for each outcome, and results were visualized on a logarithmic scale using error bar plots. For comparisons between control and experimental groups, the Mann–Whitney U test was used. A p-value < 0.05 was considered statistically significant and denoted as follows: *0.05 > *P* ≥ 0.01; **0.01 > *P* ≥ 0.001; ***P < 0.001. Results with a *P* value ≥ 0.05 were considered not statistically significant.

## Supplementary Material

Supplementary 1

MDR Reproducibility

Movies S1 to S15

science.org/doi/10.1126/science.adu2825

Figs. S1 to S10; Tables S1 to S6; MDAR Reproducibility Checklist; Movies S1 to S15

## Data Availability

All data needed to evaluate the conclusions in the paper are available in the main text or the supplementary materials. *Itga2b-GFP* mice and in-house generated antibodies are available from Bernhard Nieswandt under a material transfer agreement with the University Hospital Würzburg.
