# Supplementary material for: Platelet-derived integrin- and tetraspanin-enriched tethers exacerbate severe inflammation
Source: Science. Author manuscript; Available in PMC 2026 Jul 21. (PMC13385270; doi:10.1126/science.adu2825)
Supplement: Supplementary 1 [file NIHMS2190415-supplement-Supplementary_1.pdf]

Science AAAS

Supplementary Materials for

Platelet-derived integrin- and tetraspanin-enriched tethers exacerbate severe inflammation

Charly Kusch *et al*.

Corresponding author: Bernhard Nieswandt, bernhard.nieswandt@uni-wuerzburg.de

Science **391**, eadu2825 (2026)
DOI: 10.1126/science.adu2825

The PDF file includes:

Figs. S1 to S10
Tables S1 to S6

Other Supplementary Material for this manuscript includes the following:

MDAR Reproducibility Checklist
Movies S1 to S15

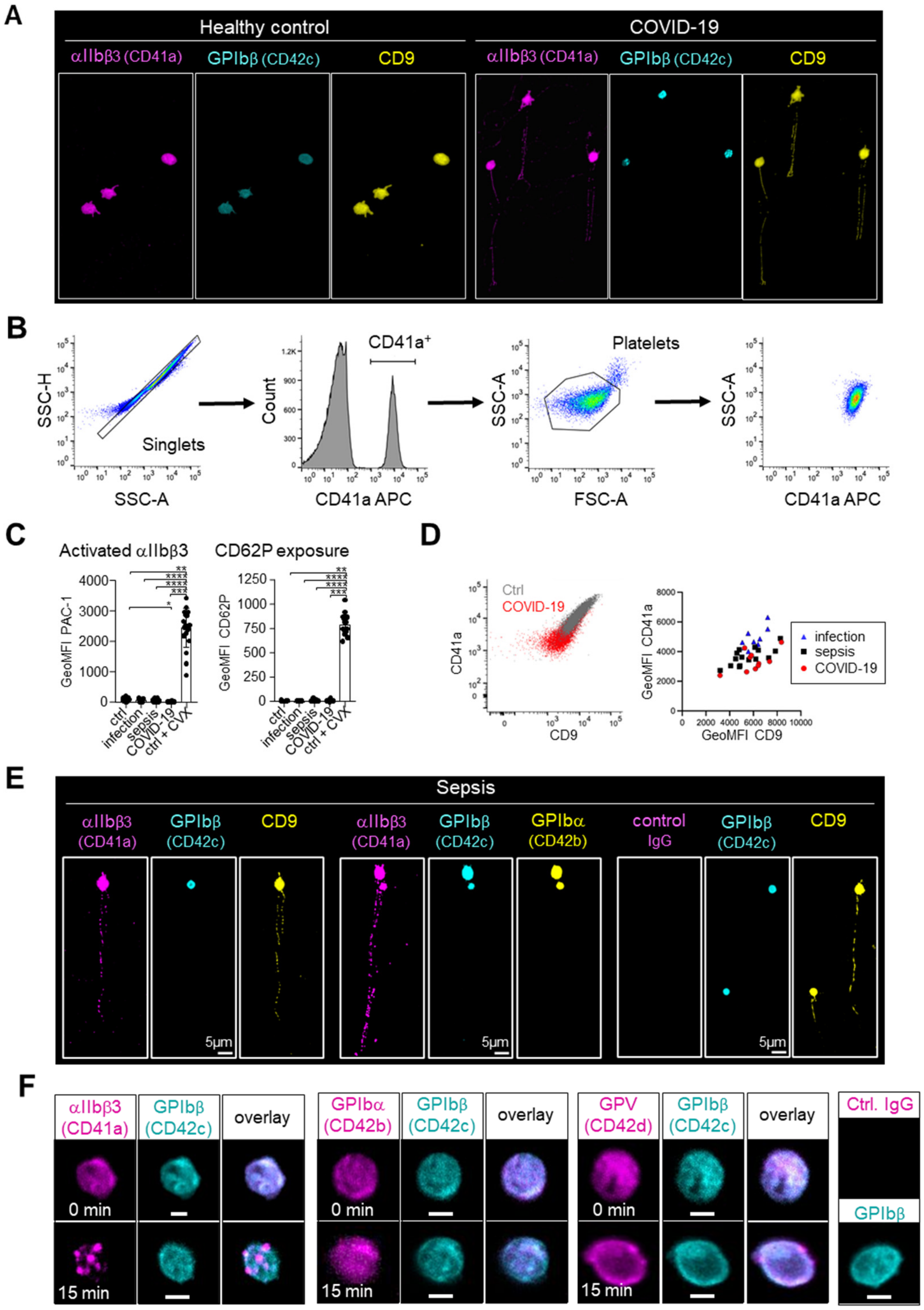

Fig. S1.

Lateral mobility of αIIbβ3 and PITT formation in human platelets. A, Immunofluorescence staining of αIIbβ3 (CD41a), GPIbβ (CD42c) and CD9 in blood smears from a healthy donor and a critically ill COVID-19 patient. Note the presence of multiple αIIb/CD9^{+} / GPIX^{-} tethers (PITTs) in the patient sample. Scale bar: 5 µm. **B,** Gating strategy used for peripheral blood platelet analysis

shown in Fig.1. **C**, CD62P exposition and αIIbβ3 activation (quantified by PAC-1 antibody binding) were determined using flow cytometry. Platelets of healthy controls were stimulated using convulxin (CVX) as positive control. Under resting conditions platelets of healthy controls and patients were not (pre-)activated. Agonist stimulation caused a marked increase of CD62P exposition and integrin activation in healthy controls. Statistical analysis: Kruskal-Wallis test. Each datapoint represents one individual measurement. *p<0.05, **p<0.01, ***p<0.001, ****p<0.0001. **D,** Flow cytometric analysis of CD41a (αIIb) and CD9 expression in platelets from patients and controls. Left panel: Representative overlay showing CD41a (y-axis) and CD9 (x-axis) dot plots of a COVID-19 patient (red) compared to a healthy control (grey). Right panel: Bivariate plot of geometric mean fluorescence intensity (GeoMFI) values for CD41a and CD9 across patient cohorts: non-sepsis infection (blue triangles), sepsis (black squares), and COVID-19 (red circles). Both markers showed a parallel reduction, with a significant positive correlation across the cohort ($r = 0.44$, $p \leq 0.01$; Pearson correlation). **E**, Immunofluorescence staining of αIIbβ3 (CD41a), GPIbβ (CD42c), CD9, and GPIbα (CD42b) in blood smears from critically ill sepsis patients. Note that the tethers (PITTs) stained positive for αIIbβ3 and CD9, but negative for GPIbα or GPIbβ. No staining was detected with the corresponding directly labeled isotype control IgGs. **F**, Maximum projections of confocal images comparing the distribution of the indicated receptors on human platelets fixed before (0 min) or after 15 min incubation with the respective fluorescently labeled mAbs (10 µg/mL). Note the pronounced clustering of αIIbβ3 by Gi5^{AF546}, whereas no clustering is observed with mAbs against GPV (LUM11^{AF647}), GPIbα (HIP1^{AF647}) or GPIbβ (p0p1^{AF488}). Scale bar: 1 µm.

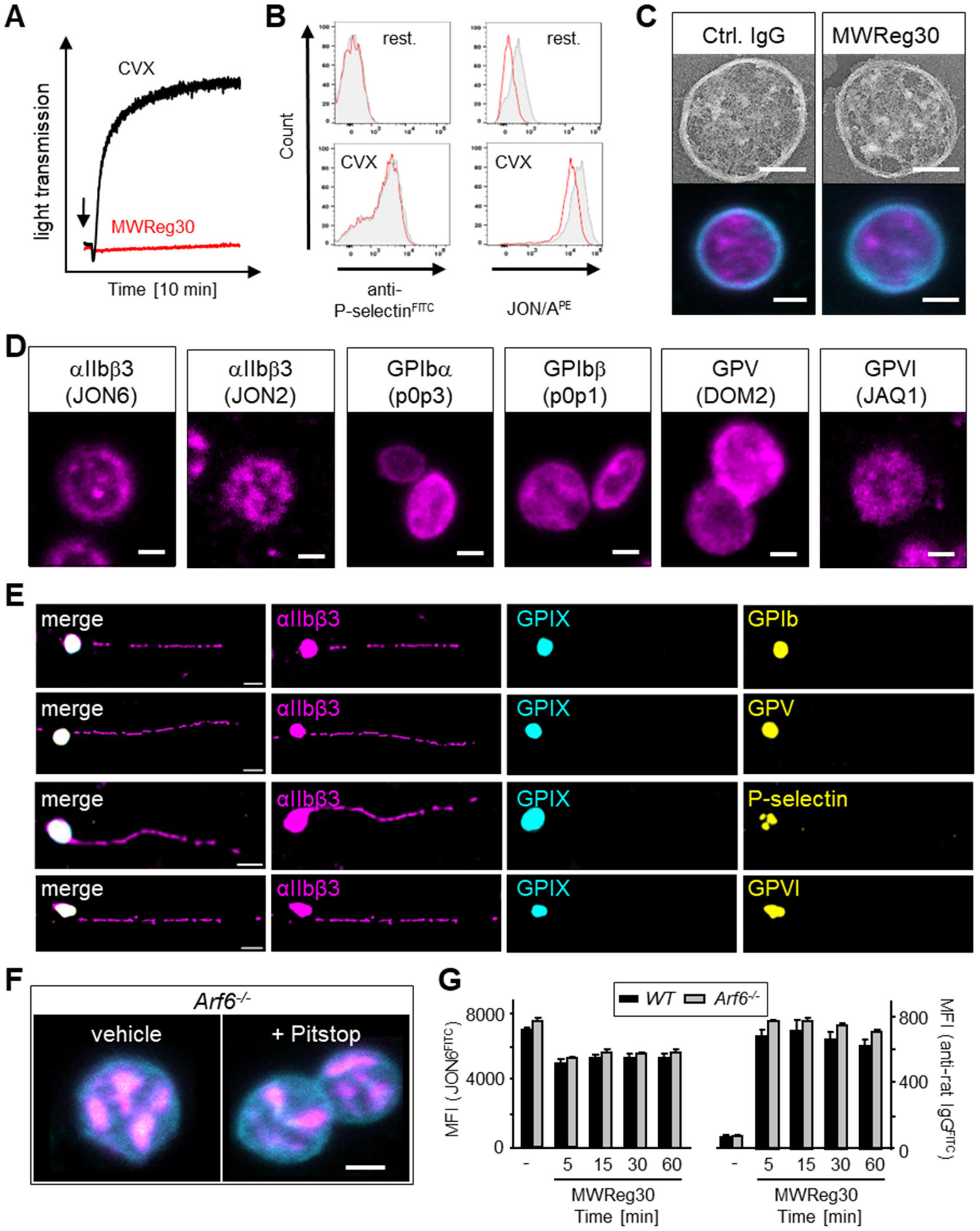

Fig. S2.

Clustering of αIIbβ3 occurs independently of platelet activation and Arf6-mediated trafficking. A, Washed mouse platelets were stimulated with 1 µg/mL convulxin (CVX) or 10 µg/mL MWReg30 (black arrow) and platelet aggregation was monitored by light transmission aggregometry. Note that MWReg30 does not induce aggregation. **B**, Washed mouse platelets were incubated with control IgG (grey histograms) or MWReg30 (10 µg/mL, red lines) and subsequently stimulated with 1 µg/mL CVX or left untreated. Platelet activation was assessed by flow cytometric analysis of P-selectin exposure (WUG.E9^{FITC}) and αIIbβ3 activation (JON/A^{PE}). **C**, Platinum replica electron microscopy (upper panels) and confocal fluorescence microscopy (lower panels)

showing unaltered distribution of actin- (magenta) and tubulin- (cyan) in MWReg30-treated platelets. Scale bar: 1 µm. **D**, Maximum intensity projections of confocal images comparing the distribution of the indicated receptors on mouse platelets fixed after 15 min incubation with the indicated antibodies. Scale bar: 1 µm. **E**, Representative images of fixed PITT-forming platelets in blood smears from LPS-treated mice (5 µg/g b.w., 4 h). Platelets were stained for αIIbβ3 ($JON6^{AF488}$, magenta) and GPIX ($p0p6^{AF546}$, cyan). Extended PITTs displayed selective enrichment of αIIbβ3, whereas GPIbβ ($p0p1^{AF647}$), GPV ($DOM2^{AF647}$), P-selectin/CD62P ($WUG1.9^{AF647}$), and GPVI ($JAQ1^{AF647}$) were absent. Images were acquired using a Thunder Imager with a 63× objective. Scale bar: 2 µm. **F**, Maximum intensity projections of confocal images of resting *Arf6*$^{fl/fl\ Pf4\text{-}cre}$ platelets showing αIIbβ3 clustering after treatment with $MWReg30^{AF647}$ (magenta) for 15 min in the presence or absence of the clathrin inhibitor Pitstop 1, counterstained with $p0p6^{AF488}$ (cyan). **G**, WT and *Arf6*$^{fl/fl\ Pf4\text{-}cre}$ platelets were incubated with 20 µg/mL MWReg30, washed, and surface abundance of αIIbβ3 (using $JON6^{FITC}$, left panel) and **G**, presence of MWReg30 on the platelet surface (using anti-rat IgG^{FITC}, right panel) were quantified by flow cytometry over time.

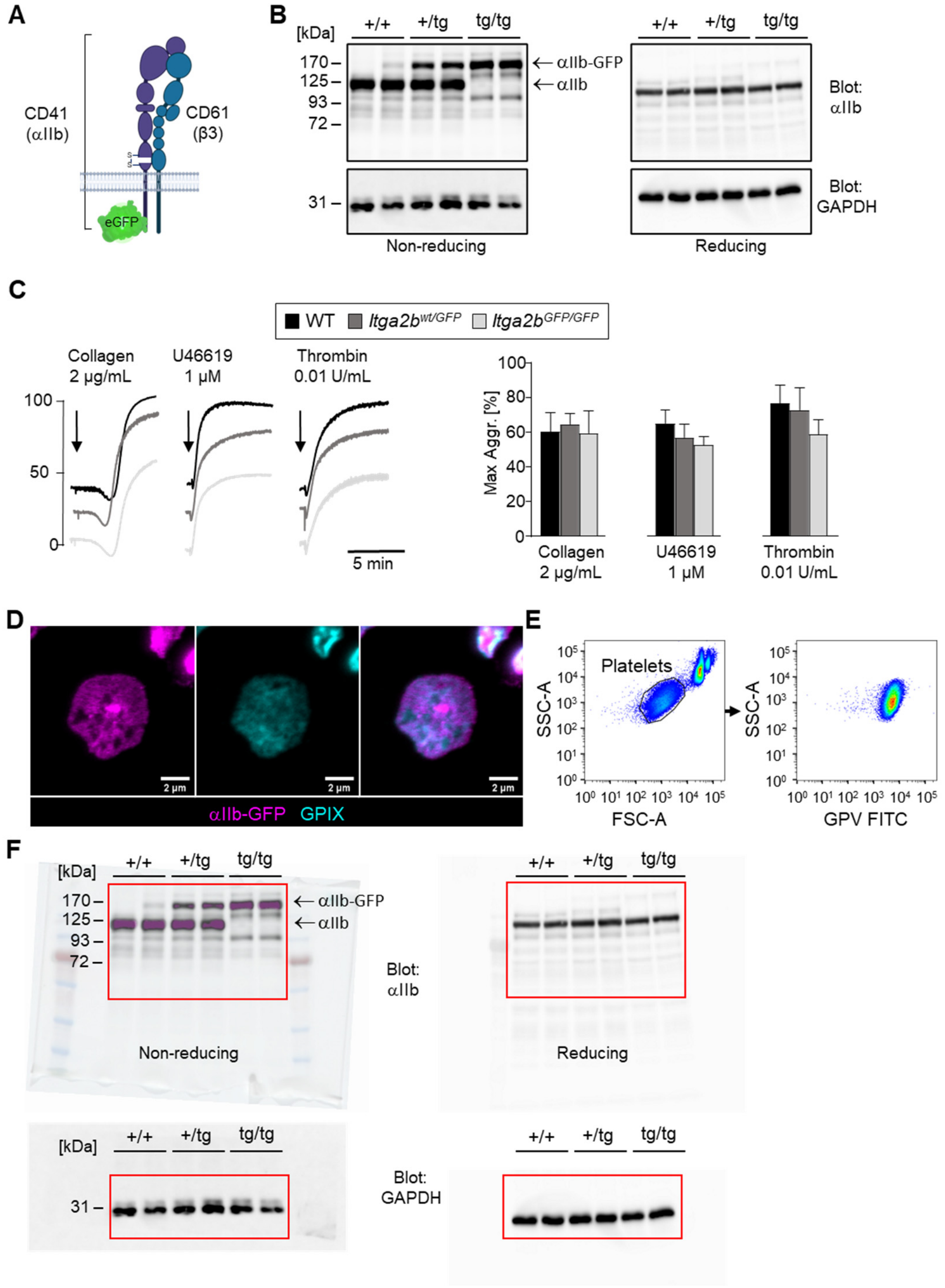

Fig. S3.

Generation of a mouse line expressing GFP-tagged integrin αIIb (CD41). A, Schematic representation of αIIbβ3 indicating the position of the C-terminal eGFP-fusion on the αIIb subunit. Note that αIIb consists of two disulfide-linked polypeptide chains. **B**, Western blot analysis of αIIb (CD41) expression in platelet lysates from wt, heterozygous (wt/tg) and homozygous *Itga2b-GFP* transgenic (tg/tg) mice – uncropped gel in **F**. **C**, Washed platelets from *wt/wt*, *wt/tg*, *tg/tg Itga2b-*

GFP mice were stimulated with the indicated agonists (black arrow) and light transmission was recorded over 10 min. Left: Representative aggregation curves. Right: Quantification of maximal aggregation in response to the indicated agonists. Data are presented as mean ± SD, n = 6. **D**, *Itga2b*$^{GFP/GFP}$ platelets were allowed to spread on fibrinogen for 20 min in the presence of 0.005 U/mL thrombin and counterstained with anti-GPIX^{AF647} (cyan). Note the homogenous distribution of αIIb-GFP (magenta) across the platelet membrane. **E**, Gating strategy used for mouse peripheral blood platelet analysis shown in this manuscript. **F**, Uncropped Western blot gels of the blots depicted in fig. S3B.

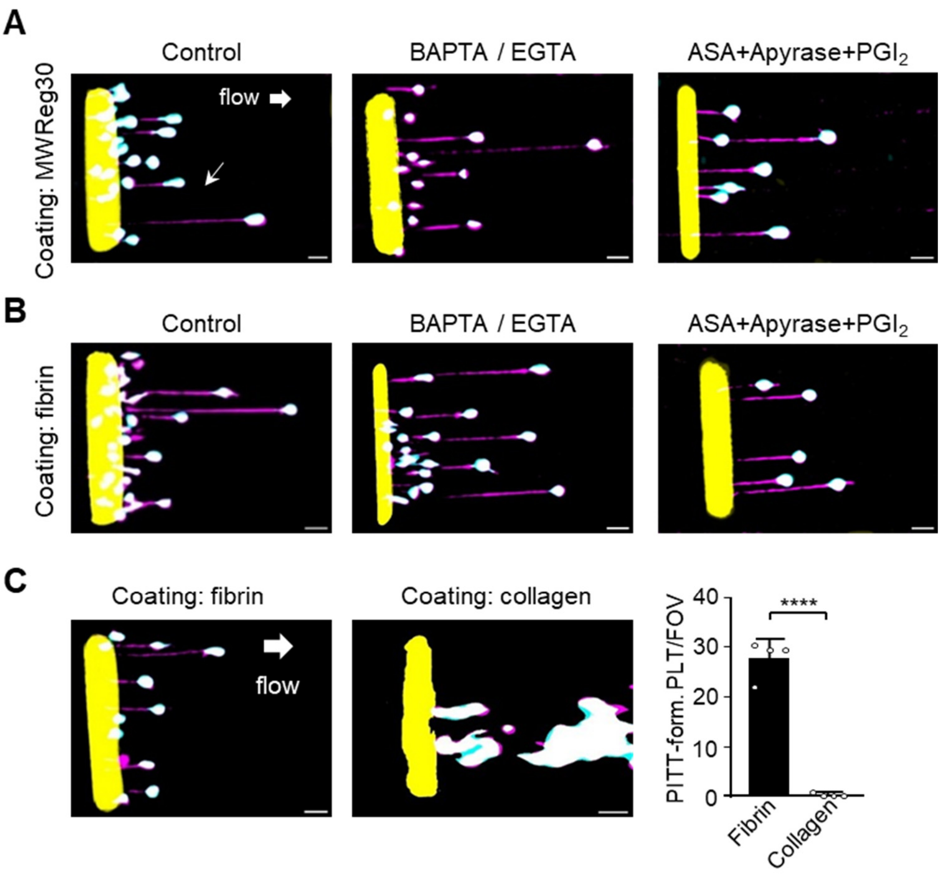

Fig. S4.

Inhibition of platelet activation does not impair PITT formation on MWReg30 or fibrin. A-B, Representative images of *Itga2b*$^{GFP/GFP}$ (αIIbβ3, magenta) platelets in whole blood, counterstained with anti-GPIX^{AF546} (cyan) during flow chamber runs over PRIMO-patterned surfaces (patterns: yellow) coated with MWReg30 (**A**) or fibrin- (**B**) at a shear rate of 1000 s^{-1} (n=6). Arrows indicate the direction of blood flow from left to right. Prior to perfusion, the blood was preincubated for 10 min with either vehicle (control), BAPTA (20 µM) + EGTA (0.5 mM), or ASA (300 µM) + apyrase (0.02 U/mL) + PGI_2 (0.1 µg/mL). (n=4). Scale bar: 5 µm. **C**, Representative images of *Itga2b*$^{GFP/GFP}$ (αIIbβ3, magenta) platelets in whole blood, counterstained with anti-GPIX^{AF546} (cyan) during flow chamber runs over PRIMO-patterned surfaces (patterns: yellow) coated with fibrin or collagen at a shear rate of 1000 s^{-1}. Arrows indicate the direction of blood flow from left to right. Scale bar: 5 µm. Data shown as mean ±SD; n=4 mice; ****p<0.0001 (Welch's t test).

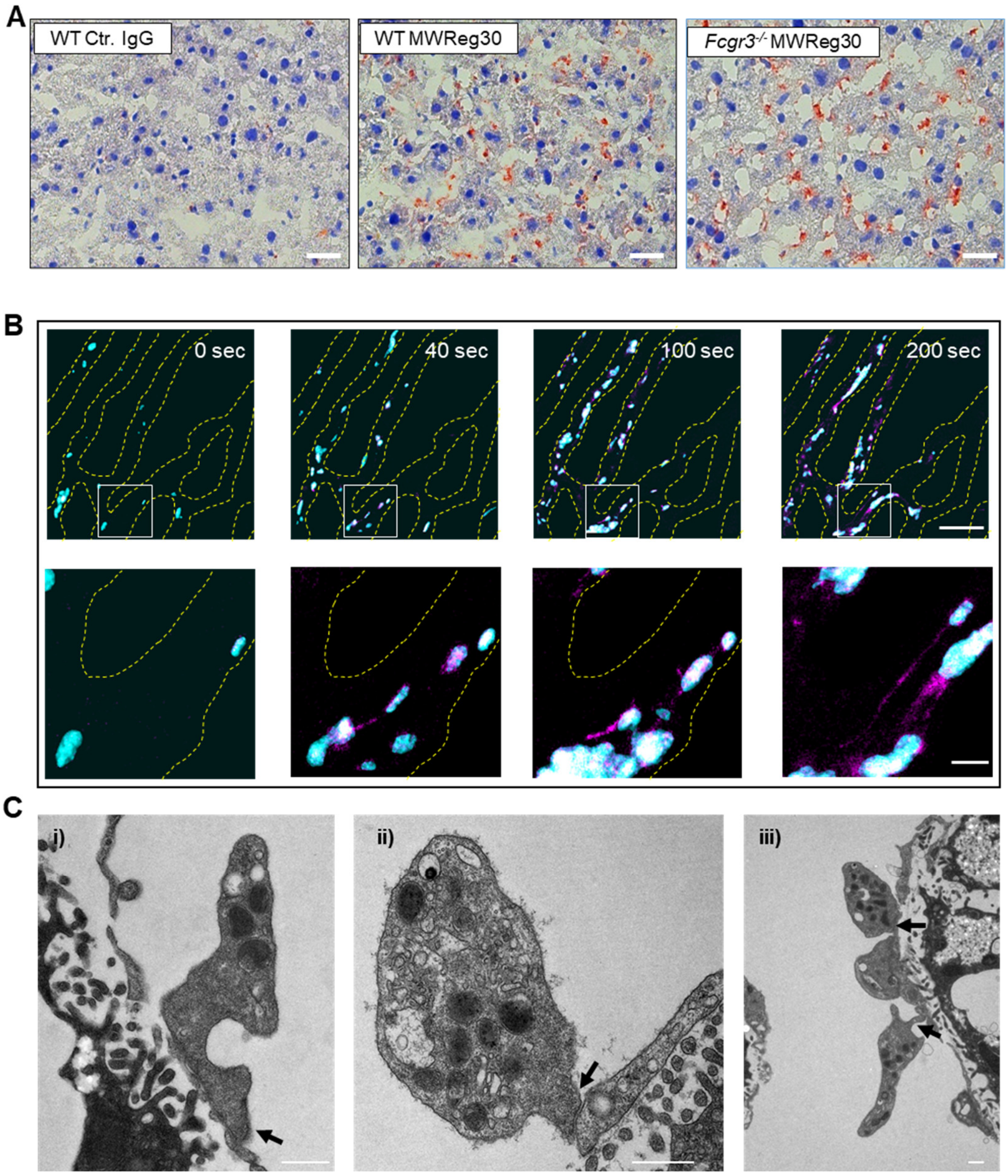

Fig. S5.

MWReg30-opsonized platelets accumulate in the liver, interact with LSECs and produce large αIIbβ3^{+}/GPIX^{-} tethers (PITTs). A, Liver cryosections from WT and *Fcgr3*$^{-/-}$ mice 30 min after intravenous injection of control IgG (WT, left) or MWReg30 (3 µg/g b.w.). Platelets were stained with HRP-conjugated anti-GPIbβ antibodies and visualized using 3-amino-9-ethylcarbazole (red); nuclei were counterstained with hematoxylin. Scale bar, 10 µm. **B**, Intravital CLSM of liver sinusoids in WT mice preinjected with anti-GPIX^{AF546} (0.2 µg/g) and anti-CD105^{AF647} (0.4 µg/g) to label platelets and liver sinusoidal endothelial cells (LSECs), respectively. Injection of MWReg30^{AF488} (0.2 µg/g b.w.) resulted in rapid accumulation of highly polarized platelets at LSECs. Most platelets extended long membrane tethers at the LSEC interface that were strongly positive for MWReg30/αIIbβ3 (magenta) but consistently negative for GPIX (cyan), resembling PITTs. Representative images are shown. See also movie 8. **C**, Transmission

electron microscopy (TEM) of PITT-forming platelets attached to LSECS in *Fcgr3*$^{-/-}$ mice 30 min after intravenous MWReg30 injection (2 µg/kg b.w.). (i, ii) Platelets in the process of PITT formation remain discoid with a regular distribution of granules and no classical signs of activation; PITTs (highlighted with arrows) are devoid of granules and mitochondria. (iii) Platelets attached to LSECs via a single PITT or by spreading. Scale bar 500 nm.

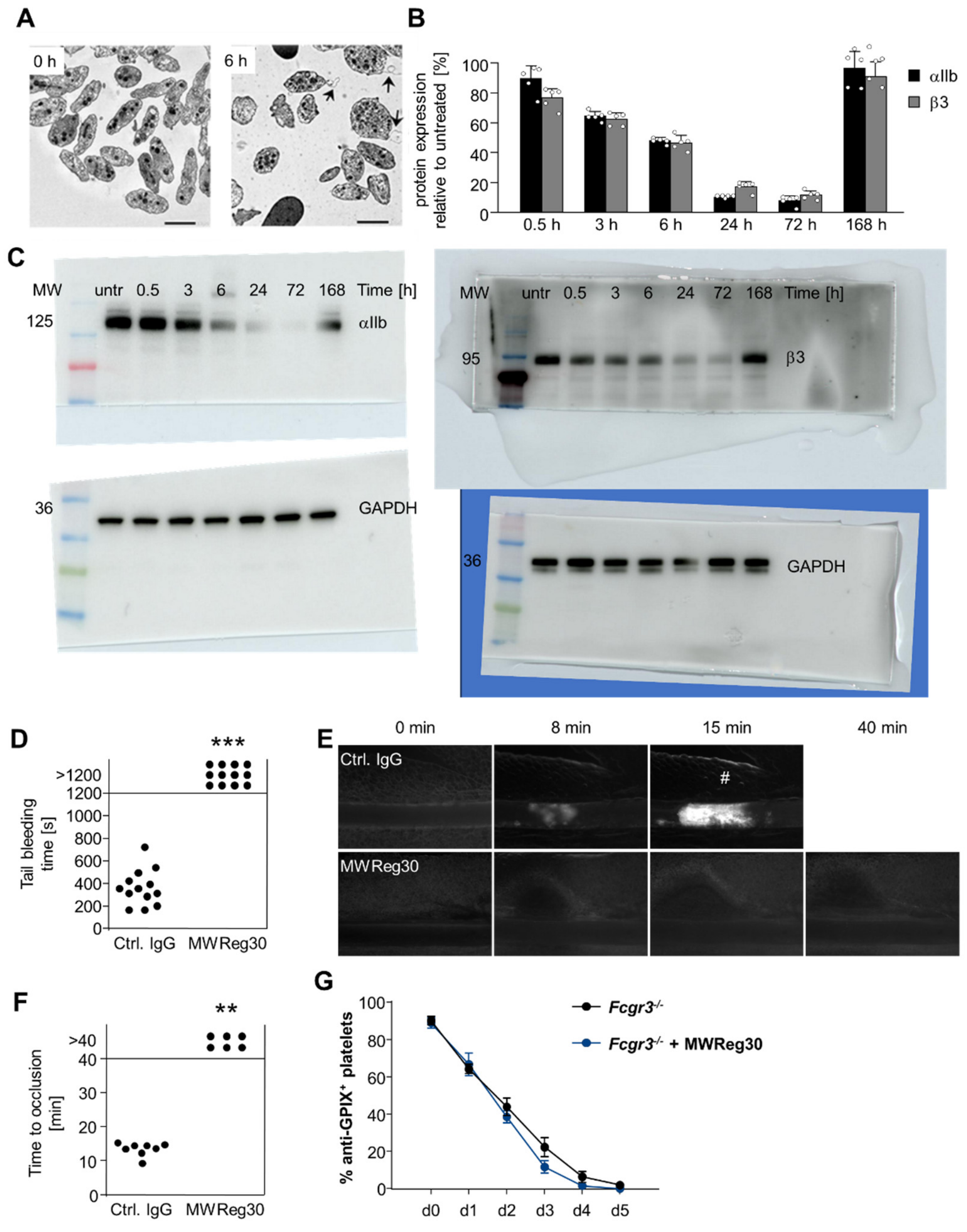

Fig. S6.

Antibody-induced αIIbβ3-depletion abrogates hemostasis and thrombosis. A, Transmission electron microscopy of control platelets (0 h, left) and platelets recirculating 6 h after intravenous injection of MWReg30 (3 µg/g body weight i.v.) into *Fcgr3*$^{-/-}$ mice (6 h, right). Recirculating platelets appeared rounder with some filaments still attached (arrows) but showed normal granule distribution, indicating a resting state. Scale bar, 1 µm. **B**, Densitometric quantification of αIIb (CD41) and β3 (CD61) expression, normalized to GAPDH and to untreated controls, from five independent Western blot experiments in platelet lysates of *Fcgr3*$^{-/-}$ mice at the indicated time points after MWReg30 injection (3 µg/g b.w.; see Fig. 3G for a representative blot). Data are presented as mean ± S.D.. **C**, Uncropped Western blots corresponding to the representative blot

shown in Fig. 3G. GAPDH served as loading control; Untr, untreated. **D**, Tail bleeding times of mice that were treated 24 h prior to the assay with control (Ctrl) IgG or MWReg30 (3 µg/g body weight i.v.). **E-F**, Thrombus formation in small mesenteric arterioles induced by topical application of 20% $FeCl_3$ in mice 24 h after injection of Ctrl IgG or MWReg30 (3 µg/g body weight i.v.). Time to occlusion and representative images are shown. The hashtag (#) indicates vessel occlusion. Each symbol represents an individual mouse. **P<0.01. **G**, *Fcgr3*$^{-/-}$ mice were injected intravenously with MWReg30 or control IgG (3 µg/g body weight). Platelet lifespan was determined by labeling with anti-GPIX^{AF488}, and the percentage of AF488^{+} platelets was measured daily by flow cytometry.

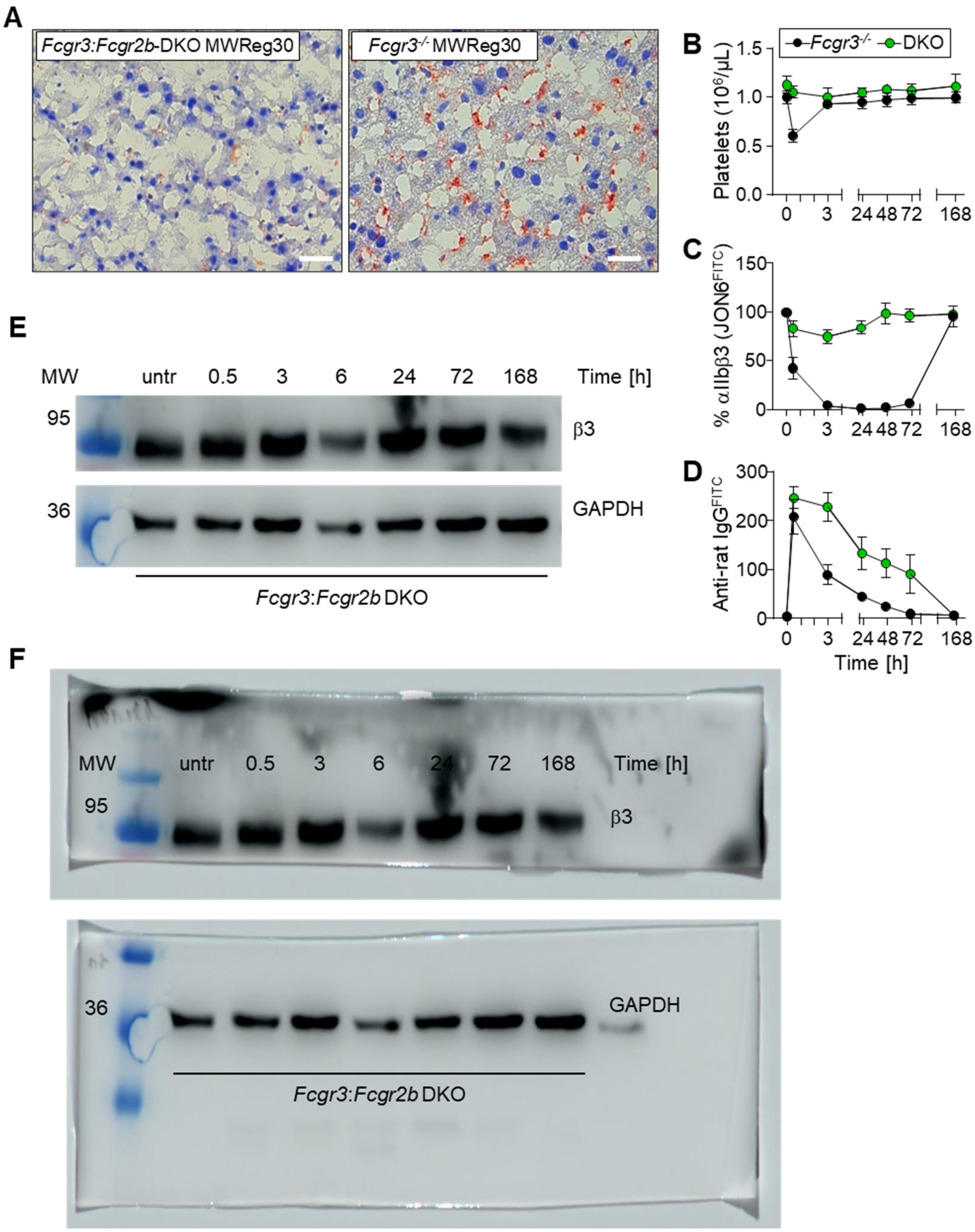

Fig. S7.

MWReg30 triggers PITT formation in vivo by anchoring on FcγRIIB. A, Detection of platelets in liver cryosections from *Fcgr3:Fcgr2b* double knockout (DKO) and *Fcgr3*$^{-/-}$ mice 30 min after i.v. injection of control IgG or MWReg30 (3 µg/g body weight). Platelets were stained with HRP-conjugated anti-GPIbβ antibodies and visualized with 3-amino-9-ethylcarbazole (red); nuclei were

counterstained with hematoxylin. Scale bar = 10 µm. **B**, Platelet counts in *Fcgr3*$^{-/-}$ (black) and *Fcgr3:Fcgr2b DKO* mice (green) were measured at the indicated time points after MWReg30 injection. **C**, Surface expression of αIIbβ3 and **D**, surface-bound MWReg30 were measured by flow cytometry using JON6^{FITC} and anti-rat IgG^{FITC} antibodies, respectively. Data are presented as mean ± SD (n=6). **E**, Western blot analysis of β3 (CD61) expression in platelet lysates from *Fcgr2b:Fcgr3* DKO mice at the indicated time points after MWReg30 injection (3 µg/g body weight), using glyceraldehyde-3-phosphate dehydrogenase (GAPDH) as loading control. Untr, untreated. **F**, shows the uncropped blot of E.

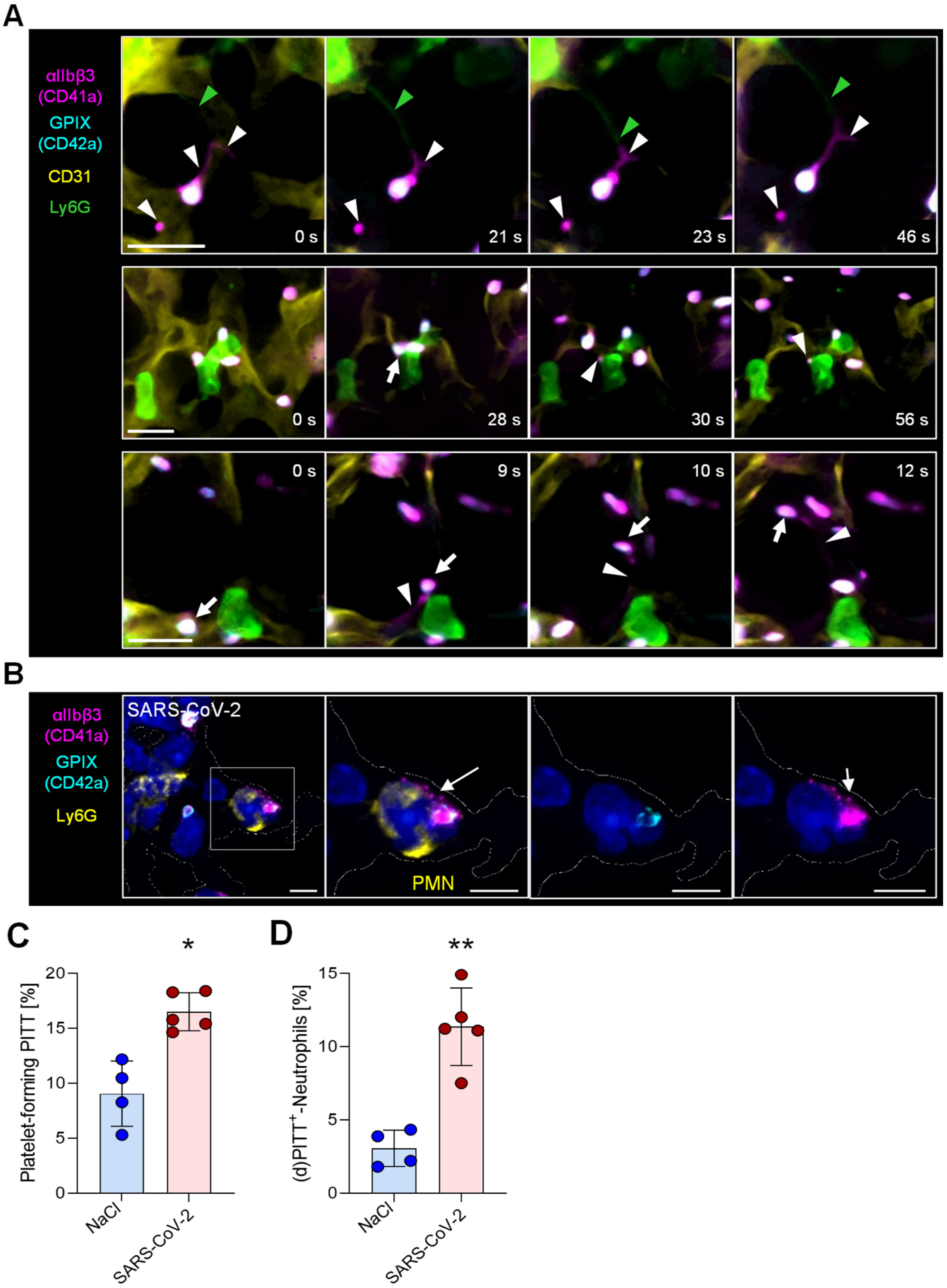

Fig. S8.

PITT formation in acute lung injury and SARS-CoV-2 infection. A, Platelets form and deposit PITTs on neutrophils and endothelial cells in vivo in a model of LPS-induced acute lung injury (ALI). Representative intravital CLSM images showing PITT and *d*PITT formation in the lungs of wild-type mice intranasally challenged with LPS (5 µg/g b.w., 4 h before IVM). Platelets were

labeled for GPIX (cyan) and αIIb (magenta); neutrophils with anti-Ly6G (green); and the vasculature with anti-CD31 (yellow). Scale bar: 5 µm. Time is indicated in seconds. White arrows mark PITT-forming platelets, white arrowheads indicate (*d*)PITTs, and green arrowheads highlight neutrophil-derived tethers. See also movie 12. **B-D**, Increased PITT formation in the lung of BALB/c mice 3 days after the exposure to aerosolized mouse-adapted SARS-CoV-2 strain. Cryosections were stained for platelet markers αIIbβ3 (magenta) and GPIX (cyan), neutrophils (anti-Ly6G, yellow), and counterstained with DAPI (blue). (*d*)PITTs (αIIbβ3+/GPIX-, white arrows). (*d*)PITT deposition on the vessel wall (C) or on neutrophils (D) was quantified. Data are expressed as a percentage of (*d*)PITTs respect to the platelet count in the analysed FOV [1 mm^2]. Significance was assessed by Mann-Withney U test and is expressed as *P < .05, ***P < .001, vs. indicated group.

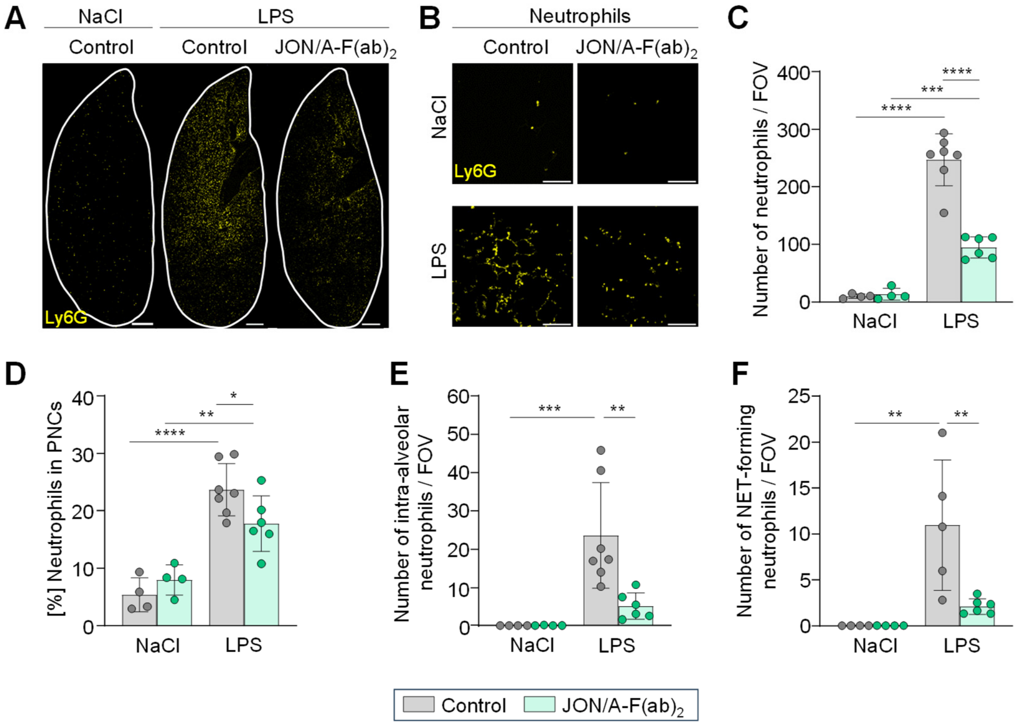

Fig. S9.

Blockade of αIIbβ3 reduces LPS-induced alveolar neutrophil recruitment and pulmonary inflammation. WT were pretreated intravenously with vehicle or JON/A-F(ab)2 (2 µg/g b.w.). After 1 hour the mice received either NaCl or LPS (10 µg/g b.w.) intranasally. Lungs were harvested 4 h later. Immunofluorescence staining was performed on cryosections of the lungs. Images were acquired using a Thunder Imager DMi8 equipped with a 20x objective. **A**, Representative micrographs of whole left lung lobes showing the extent of pulmonary neutrophil infiltration (Ly6G+, yellow). Dashed lines indicate lobe boundaries. Scale bars: 1 mm. **B**, Magnified examples of individual fields of view (FOV) used for neutrophil quantification ($Ly6G^+$, yellow, left panel). Scale bar: 80 µm. **C**, Quantification of total neutrophil numbers. **D**, Percentage of neutrophils forming platelet-neutrophil complexes (PNCs). **E**, Quantification of intra-alveolar neutrophils. **F,** Fraction of neutrophils undergoing neutrophil extracellular trap (NET) formation. Each data point represents the average neutrophil count from 10 fields of view (FOV) per mouse. Platelet aggregates were virtually undetectable in the lungs of all analyzed mice, confirming previous observations[52]. ** $P < 0.01$, *** $P < 0.001$.

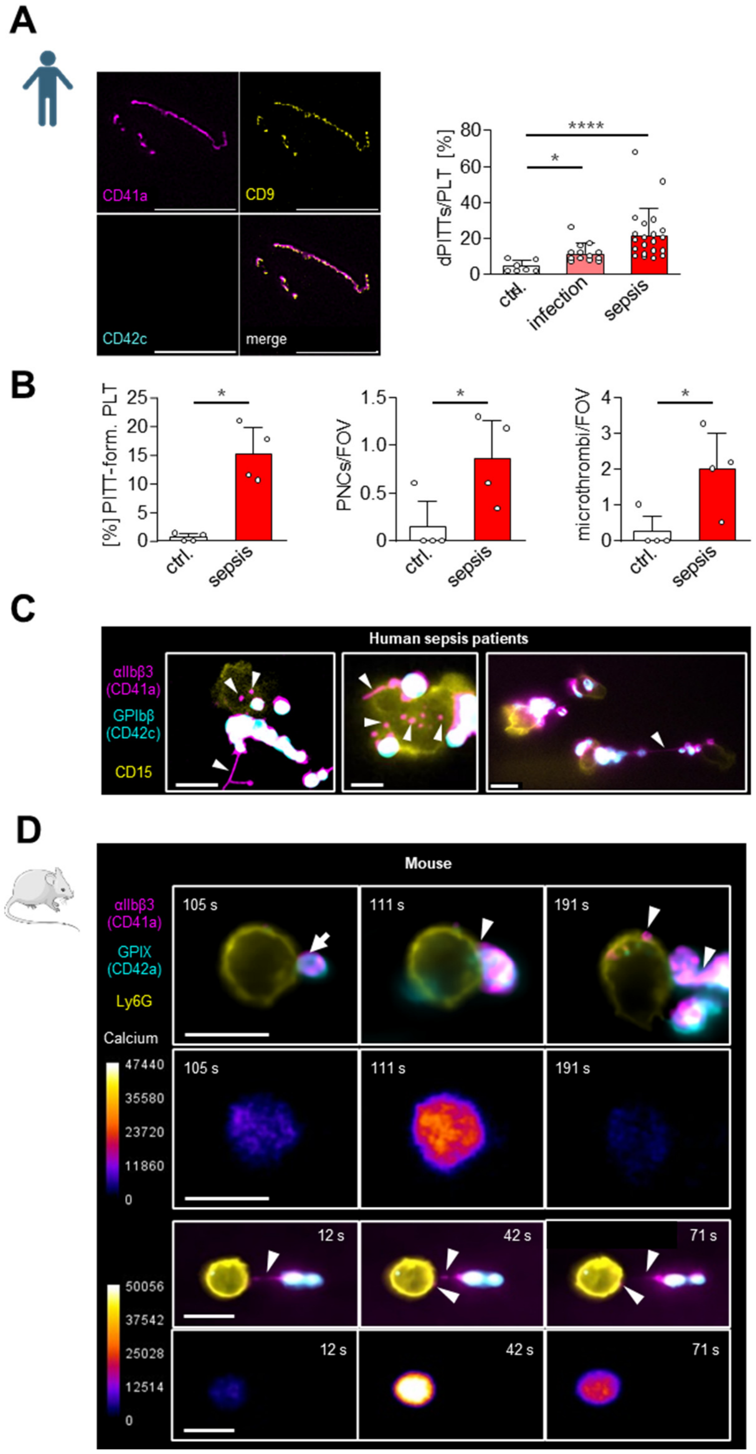

Fig. S10

PITT formation and release on neutrophils in human and mouse blood under flow on fibrin. **A**, Increased numbers of detached PITTs (dPITTs) in blood smears of sepsis patients. Samples from healthy controls (ctrl), patients with non-sepsis infection, and patients with sepsis were

stained for CD41a, CD9, and CD42c using fluorophore-conjugated antibodies and analyzed by confocal laser scanning microscopy. dPITTs were defined as $CD41a^{+}/CD9^{+}/$ $CD42c^{-}$ structures. Statistical analysis was performed using the Kruskal–Wallis test. Scale bars, 10 µm. $*p < 0.05$; $****p < 0.0001$. **B**, Quantification of PITT formation, platelet–neutrophil complexes (PNCs), and micro-thrombi. Human neutrophils from healthy donors were allowed to adhere to a homo-geneous fibrin surface, fol-lowed by perfusion of anti-coagulated whole blood from healthy donors (ctrl) or sepsis patients (sepsis) at 500 s^{-1}. Data are presented as mean ± s.d.; n = 4 donors per group; $*p < 0.05$ (Mann–Whitney U test). **C**, Representative images showing PITT formation and release on neutrophils in blood from a patient with severe sepsis. Platelets were double labeled for GPIbβ (CD42c, cyan) and αIIbβ3 (CD41a, magenta); neutrophils were stained for CD15 (yellow). **D**, Representative time-lapse images showing PITT formation and release on neutrophils in mouse blood. Bone marrow-derived neutrophils were stained with anti-Ly6G (yellow), loaded with the Ca^{2+} indicator Fluo-4 (Fire LUT), and allowed to adhere to a fibrin-coated surface for 30 min at 37 °C. Heparinized whole blood was preincubated with LPS (1 µg/mL, 30 min), double labeled for GPIX (cyan) and αIIbβ3 (magenta), and perfused at a shear rate of 500 s^{-1}. Scale bar: 5 µm. Time indicated in seconds. See also movie 15.

Table S1.

Basic patient characteristics. Units are indicated in parentheses; normal reference ranges are provided where applicable. In-hospital stay refers to the duration of inpatient treatment at the University Hospital Würzburg.
Abbreviations: ARDS, acute respiratory distress syndrome; IQR, interquartile range; BMI, body mass index; CHF, chronic heart failure; CKD, chronic kidney disease; COPD, chronic obstructive pulmonary disease; CRP, C-reactive protein; DIC, disseminated intravascular coagulation; Hb, hemoglobin; Hct, hematocrit; INR, international normalized ratio; PCT, procalcitonin; PLT, platelet; SIC, sepsis-induced coagulopathy; WBC, white blood cell count; SOFA, sequential organ failure assessment.

Groups and parameters	Infection (n=28)	Sepsis (n=39)	COVID-19 (n=18)
**Patient data and overall disease severity**			
Age (years), median (IQR)	72 (69-80)	60 (51-74)	69 (62-80)
Male gender (n)	16 (57%)	27 (69%)	7 (39%)
BMI, median (IQR)	26 (24-29)	26 (22-32)	28 (24-29)
28-day-mortality (n)	0 (0%)	7 (18%)	4 (21%)
In-hospital stay [days], median (IQR)	8 (6-16)	16 (10-37)	19 (10-30)
ARDS (n)	0 (0%)	12 (31%)	11 (61%)
DIC (DIC score ≥ 5)	0 (0%)	5 (13%)	1 (6%)
SIC (SIC score ≥ 4)	0 (0%)	19 (49%)	3 (17%)
**Comorbidity (n)**			
COPD	6 (21%)	6 (15%)	1 (6%)
Diabetes	8 (29%)	9 (23%)	4 (22%)
CKD	3 (11%)	10 (26%)	3 (17%)
CHF	7 (25%)	10 (26%)	3 (17%)
**Infection site and infectious agents (n)**			
Respiratory tract/pneumonia	10 (35%)	19 (49%)	18 (100%)
Urinary tract infection	8 (29%)	6 (15%)	0 (0%)
Other infection sites	10 (35%)	14 (36%)	0 (0%)
Gram-negative bacteria	2 (7%)	10 (26%)	/
Gram-positive bacteria	3 (11%)	3 (8%)	/
Gram-positive and -negative	0 (0%)	1 (3%)	/
Viral infection	5 (18%)	2 (5%)	18 (100%)
Unknown infectious agent	18 (64%)	23 (59%)	/

Table S1 continued:

**Groups and parameters**	**Infection (n=28)**	**Sepsis (n=39)**	**COVID-19 (n=18)**
**Laboratory values, median (IQR)**			
PLT [150-350 /nl]	236 (175-298)	155 (84-263)	215 (144-292)
WBC [5-12 /nl]	12.6 (9.2-16.7)	12.7 (8.4-17.0)	8.9 (7.1-11.6)
Hb [14-18g /dl]	11.3 (9.8-12.8	9.6 (8.4-11.2)	10.9 (9.6-12.1)
Hct [42-50 %]	33.4 (29.6-37.9)	28.8 (24.6-32.8)	31.9 (28.4-34.9)
Creatinine [0-1.17 mg/dl]	1.15 (0.99-1.56)	1.17 (0.76-2.67)	0.94 (0.74-1.37)
Bilirubin [0.1-1.2 mg/dl]	0.6 (0.4-0.8)	0.8 (0.4-1.5)	0.5 (0.3-0.6)
CRP [0-0.5 mg/dl]	12.8 (5.7-17.1)	18.5 (10.4-26.8)	13.43 (6.7-19.1)
PCT [0-0.5 ng/dl]	0.5 (0.1-5.6)	9.2 (1.4-39.7)	2.2 (0.2-4.3)
INR [0.85-1.18]	1.06 (0.99-1.15)	1.16 (1.07-1.32)	1.06 (1.00-1.08)
D-Dimers [0-0.5 ng/dl]	1.38 (0.7-3.05)	2.8 (1.22-7.69)	1.69 (0.79-4.38)
**Clinical scores, median (IQR)**			
SOFA	0 (0-1)	8 (4.5-9)	4.5 (4-7)
DIC	2 (2-3)	3 (2-4)	3 (2-3)
SIC	0.5 (0-1)	3 (2-4)	2 (2-3)
**Respiratory support (n)**			
Mechanical ventilation	0 (0%)	18 (46%)	8 (44%)

Table S2.

Blood cell counts and platelet membrane glycoprotein expression in MWReg30-treated *Fcgr3*$^{-/-}$ mice. *Fcgr3*$^{-/-}$ mice were treated intravenously with control IgG or MWReg30 (3 µg/g b.w.) and blood was collected after 24 h. Blood cell counts were analyzed using a Scilvet analyzer. Surface expression levels of the indicated platelet membrane glycoproteins were assessed by flow cytometry using FITC-conjugated antibodies. Data are presented as mean ± SD, n=5, representative of 3 independent experiments.

	**Control IgG**	**MWReg30**	**Significance**
Platelet count (10^3/µl)	986 ± 104	712 ± 33	***
MPV (fl)	6.62 ± 0.36	6.16 ± 0.22	n.s.
WBC (10^3/µl)	6.08 ± 1.10	5.50 ± 1.70	n.s.
LYM (10^3/µl)	4.16 ± 0.71	4.22 ± 0.82	n.s.
MON (10^3/µl)	0.24 ± 0.10	0.22 ± 0.12	n.s.
GRA (10^3/µl)	2.08 ± 0.56	1.66 ± 0.74	n.s.
Lymphocytes (%)	64.4 ± 6.8	67.6 ± 6.6	n.s.
Monocytes (%)	4.9 ± 0.5	4.9 ± 0.9	n.s.
RBC (10^6/µl)	9.49 ± 0.27	8.98 ± 0.42	n.s.
Hb (g/dl)	15.86 ± 0.29	15.42 ± 0.26	n.s.
HCT (%)	47.44 ± 1.74	44.12 ± 2.52	n.s.
MCV (fl)	49.00 ± 0.63	48.60 ± 1.20	n.s.
MCH (pg)	16.8 ± 0,2	16.9 ± 0.2	n.s.
**Glycoprotein expression (mean fluorescence intensity)**			
αIIbβ3	10505 ± 1284	788 ± 116	***
β3	7050 ± 567	410 ± 48	***
CD9	20797 ± 1568	9751 ± 864	***
GPIbα	6651 ± 318	6243 ± 421	n.s.
GPV	4280 ± 131	4083 ± 250	n.s.
GPIX	7922 ± 290	7682 ±249	n.s.
β1	3812 ± 418	3689 ± 283	n.s.
α2	797 ± 41	673 ± 99	n.s.
α5	2342 ± 225	2136 ± 81	n.s.
α6	5241 ± 335	4682 ± 438	n.s.
CD84	502 ± 50	488 ± 26	n.s.
CLEC-2	2670 ± 565	2441 ± 190	n.s.
GPVI	835 ± 30	748 ± 74	n.s.

Abbreviations:
MPV: Mean platelet volume
WBC: White blood cell count
LYM: Lymphocyte count
MON: Monocyte count
GRA: Granulocyte count
RBC: Red blood cell count
Hb: Hemoglobin
HCT: Hematocrit
MCV: Mean corpuscular volume
MCH: Mean corpuscular/cellular hemoglobin

Table S3.

Pearson correlation of CD41a expression with clinical parameters. The Pearson correlation coefficient (r) was calculated for the listed parameters (units are indicated in parentheses for non-binary variables). P-values were adjusted for multiple testing using the Holm–Bonferroni method; significance is indicated as follows: $P < 0.05$ (*), $P < 0.01$ (**), $P < 0.001$ (***). In-hospital stay refers to the duration of inpatient treatment at the University Hospital Würzburg.
Abbreviations: ARDS, acute respiratory distress syndrome; ASA, acetylsalicylic acid; CI, confidence interval; CRP, C-reactive protein; DIC, disseminated intravascular coagulation; INR, international normalized ratio; DOACs, direct oral anticoagulants; ns, not significant; PCT, procalcitonin; SIC, sepsis-induced coagulopathy; SOFA, sequential organ failure assessment; WBC, white blood cell count.

Groups and parameters	r	95% CI (2.5%; 97.5%)	p-value	Level of significance	Corrected p-value
**Patient characteristics**					
Age (years)	0.13	-0.08; 0.34	0.2220	ns	
Male gender	0.02	-0.23; 0.21	0.9260	ns	
Body mass index (ratio)	0.01	-0.19; 0.24	0.8332	ns	
**Disease severity and outcome**					
In-hospital stay (days)	-0,27	-0.46; -0.06	0.0115	*	0.0230
28-day mortality	-0.27	-0.45; -0.06	0.0142	*	0.0230
ARDS	-0,40	-0.57; -0.20	0.0001	***	0.0004
SOFA score	-0.39	-0.55; -0.19	0.0002	***	0.0006
**Treatment and concomitant medication**					
Mechanical ventilation	-0.33	-0.50; -0.12	0.0023	**	0.0046
Catecholamins	-0.27	-0.45; -0.05	0.0144	*	0.0144
ASA	-0.05	-0.26; 0.16	0.6447	ns	
Enoxaparin	-0.13	-0.34; 0.08	0.2296	ns	
Fondaparinux	-0.04	-0.25; 0.17	0.7173	ns	
Heparin	0.03	-0.19; 0.24	0.8102	ns	
DOACs	0.14	-0.07; 0.35	0.1920	ns	
Piperacillin/tazobactam	-0.15	-0.35; 0.06	0.1618	ns	
Aciclovir	-0.07	-0.28; 0.14	0.5132	ns	
Clarithromycin	-0.03	-0.24; 0.18	0.7830	ns	
Other antibiotics	0.01	-0.21; 0.22	0.9621	ns	

To be continued

Groups and parameters	r	95% CI (2.5%; 97.5%)	p-value	Level of significance	Corrected p-value
**Hemostasis and coagulation**					
Platelet count (ng/L)	0.09	-0.12; 0.30	0.4012	ns	
INR (ratio)	-0.08	-0.29; 0.13	0.4440	ns	
Fibrinogen (g/L)	-0.03	-0.25; 0.20	0.7948	ns	
D-dimers (mg/L)	-0.37	-0,55; -0.16	0.0008	***	0.0016
SIC score (value)	-0.40	-0.56; -0.20	0.0002	***	0.0006
SIC (SIC score ≥ 4)	-0.21	-0.41; -0.00	0.050	ns	
DIC score (value)	-0.37	-0.56; -0.15	0.0014	**	0.0016
DIC (DIC score ≥ 5)	-0.10	-0.32; 0.10	0.4093	ns	
**Infection sites and infectious agents**					
Respiratory tract/pneumonia	-0.07	-0.28; 0.15	0.5356	ns	
Urinary tract infection	-0.05	-0.26; 0.16	0.6484	ns	
Other infection sites	0.12	-0.10; 0.32	0.2880	ns	
Gram-negative bacteria	0.03	-0.19; 0.24	0.7973	ns	
Gram-positive bacteria	0.00	-0.21; 0.21	0.9901	ns	
Viral infection	-0.05	-0.26; 0.16	0.6279	ns	
Unknown infectious agent	0.03	-0.18; 0.24	0.7711	ns	
**Main laboratory parameters**					
WBC (n/µL)	0.09	-0.13; 0.29	0.4353	ns	
Hemoglobin (g/L)	0.29	0.09; 0.48	0.0066	**	0.0066
Hematocrit (%)	0.19	-0.03; 0.39	0.0897	ns	
Creatinin (mg/dL)	-0.07	-0.28; 0.15	0.5368	ns	
Bilirubin (mg/dL)	0.02	-0.22; 0.25	0.9008	ns	
CRP (mg/dL)	-0.17	-0.37; 0.05	0.1301	ns	
PCT (ng/mL)	-0.19	-0.40; 0.04	0.1012	ns	

Table S4.

Spearman's rank correlation of CD41a expression with clinical parameters. Spearman's rank correlation coefficient (ρ) was calculated for the listed parameters (units are indicated in parentheses for non-binary variables). P-values were grouped and corrected for multiple comparisons using the Holm–Bonferroni method; statistical significance is denoted as follows: $P < 0.05$ (*), $P < 0.01$ (**), $P < 0.001$ (***), $P < 0.0001$ (****). In-hospital stay refers to the duration of inpatient treatment at the University Hospital Würzburg.
Abbreviations: ARDS, acute respiratory distress syndrome; ASA, acetylsalicylic acid; CI, confidence interval; CRP, C-reactive protein; DIC, disseminated intravascular coagulation; INR, international normalized ratio; DOACs, direct oral anticoagulants; ns, not significant; PCT, procalcitonin; SIC, sepsis-induced coagulopathy; SOFA, sequential organ failure assessment; WBC, white blood cell count.

Groups and parameters	ρ	95% CI (2.5%; 97.5%)	p-value	Level of significance	Corrected p-value
**Patient characteristics**					
Age (years)	0.20	-0.02; 0.40	0.0729	ns	
Male gender	0.01	-0.18; 0.29	0.9260	ns	
Body mass index (ratio)	0.06	-0.18; 0.29	0.6115	ns	
**Disease severity and outcome**					
In-hospital stay (days)	-0.33	-0.51; -0.11	0.0024	**	0.0048
28-day mortality	-0.28	-0.47; -0.06	0.0096	**	0.0096
ARDS	-0.39	-0.56; -0.18	0.0003	***	0.0012
SOFA-score	-0.38	-0.56; -0.18	0.0003	***	0.0012
**Treatment and concomitant medication**					
Mechanical ventilation	-0.32	-0.50; -0.10	0.0032	**	0.0032
Catecholamins	-0.21	-0.41; 0.01	0.0545	ns	
ASA	-0.02	-0.23; 0.20	0.8909	ns	
Enoxaparin	-0.14	-0.35; 0.09	0.2150	ns	
Fondaparinux	-0.03	-0.25; 0.19	0.7619	ns	
Heparin	0.01	-0.21; 0.23	0.9054	ns	
DOACs	0.15	-0.08; 0.35	0.1809	ns	
Piperacillin/tazobactam	-0.19	-0.39; 0.03	0.0880	ns	
Aciclovir	-0.10	-0.31; 0.12	0.3516	ns	
Clarithromycin	-0.03	-0.25; 0.19	0.7620	ns	
Other antibiotics	0.07	-0.15; 0.29	0.5130	ns	

To be continued

Groups and parameters	ρ	95% CI (2.5%; 97.5%)	p-value	Level of significance	Corrected p-value
**Hemostasis and coagulation**					
Platelet count (ng/L)	0.10	-0.12; 0.31	0.3572	ns	
INR (ratio)	-0.08	-0.30; 0.14	0.4577	ns	
Fibrinogen (g/L)	-0.02	-0.25; 0.21	0.8839	ns	
D-dimers (mg/L)	-0.45	-0.61; -0.24	< 0.0001	****	0.0003
SIC score (value)	-0.38	-0.55; -0.17	0.0004	***	0.0008
SIC (SIC score ≥ 4)	-0.19	-0.40; -0.03	0.0756	ns	0.0280
DIC score (value)	-0.37	-0.56; -0.14	0.0015	**	0.0015
DIC (DIC score ≥ 5)	-0.09	-0.32; 0.10	0.4548	ns	
**Infection sites and infectious agents**					
Respiratory tract/pneumonia	-0.10	-0.31; 0.13	0.3799	ns	
Urinary tract infection	-0.06	-0.28; 0.16	0.5647	ns	
Other infection sites	0.16	-0.06; 0.37	0.1468	ns	
Gram-negative bacteria	0.03	-0.19; 0.24	0.8087	ns	
Gram-positive bacteria	0.03	-0.19; 0.25	0.7998	ns	
Viral infection	-0.04	-0.26; 0.18	0.7092	ns	
Unknown infectious agent	0.01	-0.21; 0.23	0.9098	ns	
**Main laboratory parameters**					
WBC (n/µL)	0.22	0.01; 0.42	0.0388	*	0.0388
Hemoglobin (g/L)	0.29	0.08; 0.48	0.0069	**	0.0207
Hematocrit (%)	0.25	0.04; 0.45	0.0199	*	0.0398
Creatinin (mg/dL)	0.03	-0.19; 0.25	0.7909	ns	
Bilirubin (mg/dL)	0.15	-0.10; 0.38	0.2178	ns	
CRP (mg/dL)	-0.14	-0.35; 0.08	0.1871	ns	
PCT (ng/mL)	-0.09	-0.31; 0.15	0.4545	ns	

Table S5.

Mouse strains used in this study. Overview of all wild-type, knockout, conditional knockout, and transgenic mouse lines employed, including genetic background, source/reference, and specific experimental use.

Strain	Name	species	source	RRID
C57BL/6JRj	C57Bl/6J	mouse	in-house animal facility or Charles River Laboratories	RRID:MGI:2670020
vWF$^{-/-}$	*Vwf*^{tm1Wgr}	mouse	in-house animal facility	RRID:MGI:2153009
Fcgr2b$^{-/-}$	*Fcgr2b*^{tm1Ttk}	mouse	in-house animal facility	RRID:MGI:1857166
Fcgr3$^{-/-}$	*Fcgr3*^{tm1Ttk}	mouse	in-house animal facility	RRID:3620982
Arf6$^{fl/fl}$	*Arf6*$^{tm1.1Gdp/J}$	mouse	in-house animal facility (originally from Jackson Laboratory - #028669)	RRID:IMSR_JAX:028669
Fcgr3:Fcgr2b-DKO mice	*Fcgr2b*^{tm1Ttk}/ *Fcgr3*^{tm1Ttk}	mouse	in-house animal facility	
PF4-Cre	*Tg(Pf4-icre)*^{Q3Rsko}	mouse	in-house animal facility	RRID:MGI:3764698
Itga2b-GFP	newly generated	mouse	in-house animal facility	

Table S6.

Antibodies used in this study. Overview of all antibodies applied in the experiments.

Antigen	Reactivity	Source	Clone	Accession	RRID/Reference
CD105	mouse	in-house	MJ7/18	n/a	AB_3172711
CD11b	human	BD Biosciences	ICRF44	555388	AB_395789
CD15	human	BD Biosciences	HIM1	555401	AB_395801
CD184	human	BD Biosciences	FN50	557756	AB_396862
CD41	human	BD Biosciences	HIP8	559777	AB_398671
CD41	mouse	Santa Cruz	polyclonal	sc-15328	AB_10166176
CD41a	human	BD Biosciences	PAC-1	340507	AB_2230769
CD42a/GPIX	human	BD Biosciences	Beb1	340537	AB_400050
CD42a/GPIX	mouse	in-house	p0p6	n/a	PMID: 28743899
CD42a/GPIX	mouse	Emfret Analytics	X488	X488	AB_2890921
CD42a/GPIX	mouse	in-house	Xia.B4	M051-1	AB_2827529
CD42b/GPIbα	mouse	in-house	Xia.G5	M040-1	AB_2827527
CD42c/GPIbβ	mouse/human	in-house	p0p1	n/a	PMID: 10648400
CD42d/GPV	human	in-house	LUM11	n/a	PMID: 37206993
CD42d/GPV	mouse	in-house	DOM1	n/a	PMID: 10648400
CD62L	human	BioLegend	DREG-56	304830	AB_2629555
CD62P (P-selectin)	mouse	Emfret Analytics	Wug.E9	M130-1	AB_2890922
CD66b	human	BioLegend	G10F5	305108	AB_2077855
CD84	mouse	in-house	JER1	n/a	PMID: 23025437
CD9	human	BD Biosciences	M-L13	555371	AB_395773
CD9	mouse	in-house	ULF1	n/a	PMID: 11001906
CLEC-2	mouse	in-house	INU1	n/a	PMID: 19641185
FcγRIIb	mouse	Cell Signaling	D8F9C	96397	AB_2800262
GAPDH	mouse/human	Sigma-Aldrich	polyclonal	G9545	RRID:AB_796208
GPVI	mouse	in-house	JAQ1	n/a	AB_2827531
Ly6G	mouse	BioLegend	1A8	127649	AB_2572001
vWF	human	Agilent/Dako	polyclonal	A0082	AB_2315602
α2	mouse	in-house	LEN1	n/a	PMID: 10825177
α5	mouse	in-house	BAR1	n/a	PMID: 12893753
αIIbβ3	mouse	in-house	JON6	n/a	PMID: 11001906
αIIbβ3	mouse	in-house	JON2	n/a	PMID: 11001906
αIIbβ3	human	in-house	Gi5	n/a	
αIIbβ3	mouse	Emfret Analytics	JON/A	M023-2	AB_2833084
αIIbβ3	mouse	in-house	MWReg30	n/a	AB_2833085
β1	mouse	BD Biosciences	Ha2/5	555005	AB_395639
β3	mouse/human	in-house	EDL1	n/a	PMID: 11001906
β3	mouse	Emfret Analytics	Luc.H11	M031	AB_2827528
β-actin	mouse/human	Sigma-Aldrich	polyclonal	A2066	AB_10063060

Movie S1.
Confocal time-lapse microscopy of wild-type murine platelets (pre-labeled with anti-$GPIX^{CF568}$, cyan), showing rapid αIIbβ3 clustering upon addition of $MWReg30^{AF488}$ (10 µg/mL, magenta) as indicated.

Movie S2.
Time-lapse confocal imaging of Pitstop-pretreated *Arf6*$^{-/-}$ platelets (pre-labeled with anti-$GPIX^{CF568}$, cyan), demonstrating that $MWReg30^{AF488}$-induced αIIbβ3 clustering persists even when clathrin is inhibited and Arf6-mediated endocytosis and trafficking is abolished. $MWReg30^{AF488}$ (10 µg/mL) was added as indicated.

Movie S3.
Formation of Platelet-derived Integrin & Tetraspanin-enriched Tethers (PITTs) occurs in *Itga2b*$^{GFP/GFP}$ platelets (magenta) counterstained with anti-$GPIX^{AF647}$ (cyan), during flow over vWF-coated PRIMO micropatterns (yellow) at a shear rate of 1000 s^{-1} in the presence of control Fab. Note the selective accumulation of αIIbβ3, but not GPIX, within the tethers.

Movie S4.
Blockade of GPIbα with p0p/B-Fab fragments prevents platelet adhesion to vWF and thereby abrogates the formation of Platelet-derived Integrin & Tetraspanin-enriched Tethers (PITTs). *Itga2b*$^{GFP/GFP}$ platelets (magenta), counterstained with anti-$GPIX^{AF647}$ (cyan), were perfused over vWF-coated PRIMO patterns (yellow) at a shear rate of 1000 s^{-1} in the presence of p0p/B.

Movie S5.
Blockade of αIIbβ3 by JON/A-F(ab')$_2$ fragments inhibits PITT formation without affecting platelet adhesion to vWF. *Itga2b*$^{GFP/GFP}$ platelets (magenta), counterstained with anti-$GPIX^{AF647}$ (cyan), were perfused over vWF-coated PRIMO patterns (yellow) at 1000 s^{-1} in the presence of JON/A.

Movie S6.
Time-lapse imaging of *Itga2b*$^{GFP/GFP}$ platelets (magenta), counterstained with anti-$GPIX^{AF546}$ (cyan), during flow over MWReg30-coated PRIMO micropatterns (yellow) at a shear rate of 1000 s^{-1}. PITTs form at the platelet rear edge; in some cases, the platelet detaches and leaves a stable *d*PITTs behind.

Movie S7.
As in Movie 6, time-lapse imaging of *Itga2b*$^{GFP/GFP}$ platelets (magenta), counterstained with anti-$GPIX^{AF546}$ (cyan), perfused over MWReg30-coated PRIMO patterns (yellow) at 1000 s^{-1} reveals PITT formation and, in some cases, the detachment of the parent platelet, which leaves *d*PITTs behind.

Movie S8.

WT mice were treated with MWReg30^{AF488} (1 µg/g i.v.; magenta), and liver sinusoids were visualized immediately by intravital confocal laser scanning microscopy. Platelets were counterstained with anti-GPIX^{AF546} (0.2 µg/g; cyan), and the vessel bed (stained with anti-CD105^{AF647}) is highlighted by yellow lines. The video shows platelet accumulation and attachment to LSECs 5 minutes after MWReg30^{AF488} injection. Note the widespread formation of αIIbβ3^{+}/GPIX^{-} tethers on the LSEC surface.

Movie S9-S11.

WT platelets were double-labeled in vitro with MWReg30^{AF488} (magenta) and anti-GPIX^{AF546} (cyan) for 10 min and transfused into *Fcgr3*$^{-/-}$ mice. Liver sinusoids were visualized immediately by intravital confocal laser scanning microscopy. The vessel bed (stained with anti-CD105^{AF647}) is highlighted by dashed yellow lines. The video shows platelets forming αIIbβ3-enriched tethers forming at the platelet-LSEC interface before αIIbβ3-depleted platelets detache and return to circulation.

Movie S12

Representative intravital confocal laser scanning microscopy (CLSM) of the lung 4 hours after intranasal LPS administration (5 µg/g body weight), illustrating PITT formation and deposition on neutrophils and endothelial cells within the pulmonary microvasculature. Platelets were labeled for GPIX (cyan) and αIIbβ3 (magenta); neutrophils: Ly6G (green); vasculature: CD31 (yellow). Scale bar: 5 µm. Time is indicated in minutes:seconds.

Movie S13-S14

Time-lapse confocal microscopy of PITT formation and deposition on neutrophils in human blood. Neutrophils were isolated from healthy donors, stained for CD15 (yellow), loaded with the calcium indicator Fluo-4/AM (Fire LUT) and allowed to adhere to fibrin for 30 min at 37 °C. Whole blood from the same donor was stained for GPIbβ (CD42c, cyan) and αIIbβ3 (CD41/CD61, magenta), and then perfused at 500 s^{-1} to visualize platelet-neutrophil interactions. Scale bar: 5 µm. Time is shown in minutes:seconds.

Movie S15

Time-lapse microscopy of PITT formation and deposition on neutrophils in mouse blood. Bone marrow-derived neutrophils were labeled with anti-Ly6G (yellow), loaded with the Ca^{2+} indicator Fluo-4/AM (Fire LUT), and allowed to adhere to fibrin for 30 min at 37 °C. Heparinized mouse blood was preincubated with LPS (1 µg/mL, 30 min), stained for GPIX (cyan) and αIIbβ3 (magenta), and perfused over the adherent neutrophils at a shear rate of 500 s^{-1}. Scale bar: 5 µm. Time is shown in minutes:seconds.
